# Identification of potential therapeutic intervening targets by *in-silico* analysis of nsSNPs in preterm birth-related genes

**DOI:** 10.1371/journal.pone.0280305

**Published:** 2023-03-07

**Authors:** Muhammad Bilal Azmi, Waqasuddin Khan, M. Kamran Azim, Muhammad Imran Nisar, Fyezah Jehan

**Affiliations:** 1 Department of Biochemistry, Dow Medical College, Dow University of Health Sciences, Karachi, Pakistan; 2 Department of Biosciences, Faculty of Life Sciences, Mohammad Ali Jinnah University, Karachi, Pakistan; 3 Biorepositroy and Omics Research Group, Department of Pediatrics and Child Health, Faculty of Health Sciences, Medical College, The Aga Khan University, Karachi, Pakistan; 4 Department of Pediatrics and Child Health, Faculty of Health Sciences, Medical College, The Aga Khan University, Karachi, Pakistan; 5 CITRIC Center for Bioinformatics and Computational Biology, Department of Pediatrics and Child Health, Faculty of Health Sciences, Medical College, The Aga Khan University, Karachi, Pakistan; ICMR-Regional Medical Research Centre Bhubaneswar, INDIA

## Abstract

Prematurity is the foremost cause of death in children under 5 years of age. Genetics contributes to 25–40% of all preterm births (PTB) yet we still need to identify specific targets for intervention based on genetic pathways. This study involved the effect of region-specific non-synonymous variations and their transcript level mutational impact on protein functioning and stability by various *in-silico* tools. This investigation identifies potential therapeutic targets to manage the challenge of PTB, corresponding protein cavities and explores their binding interactions with intervening compounds. We searched 20 genes coding 55 PTB proteins from NCBI. Single Nucleotide Polymorphisms (SNPs) of concerned genes were extracted from ENSEMBL, and filtration of exonic variants (non-synonymous) was performed. Several *in-silico* downstream protein functional effect prediction tools were used to identify damaging variants. Rare coding variants were selected with an allele frequency of ≤1% in 1KGD, further supported by South Asian ALFA frequencies and GTEx gene/tissue expression database. CNN1, COL24A1, IQGAP2 and SLIT2 were identified with 7 rare pathogenic variants found in 17 transcript sequences. The functional impact analyses of rs532147352 (R>H) of CNN1 computed through PhD-SNP, PROVEAN, SNP&GO, PMut and MutPred2 algorithms showed impending deleterious effects, and the presence of this pathogenic mutation in CNN1 resulted in large decrease in protein structural stability (ΔΔG (kcal/mol). After structural protein identification, homology modelling of CNN1, which has been previously reported as a biomarker for the prediction of PTB, was performed, followed by the stereochemical quality checks of the 3D model. Blind docking approach were used to search the binding cavities and molecular interactions with progesterone, ranked with energetic estimations. Molecular interactions of CNN1 with progesterone were investigated through LigPlot 2D. Further, molecular docking experimentation of CNN1 showed the significant interactions at S102, L105, A106, K123, Y124 with five selected PTB-drugs, Allylestrenol (-7.56 kcal/mol), Hydroxyprogesterone caproate (-8.19 kcal/mol), Retosiban (-9.43 kcal/mol), Ritodrine (-7.39 kcal/mol) and Terbutaline (-6.87 kcal/mol). Calponin-1 gene and its molecular interaction analysis could serve as an intervention target for the prevention of PTB.

## Introduction

Preterm birth (PTB) defined as birth before 37 weeks of gestation is the leading cause of neonatal morbidity and mortality worldwide [1]. Spontaneous Preterm Birth, accounts for approximately 70% of the preterm deliveries, and *Indicated or Iatrogenic PTB* accounts for the remaining 25% of preterm deliveries [2]. Spontaneous PTB is the result of multiple pathologic processes, including infection/inflammation, uterine ischemia, uterine over distension, abnormal allograft reaction, allergy, cervical insufficiency, and hormonal disorders [3]. Previous history of spontaneous PTB is an important risk factor of spontaneous PTB in future pregnancies, described as delivery of a liveborn before 37 weeks of gestation as result of preterm premature rupture of membranes (PROM) or spontaneous preterm labor [3, 4]. Iatrogenic PTB is the result of a medical intervention due to a fetal and/or maternal condition (e.g., fetal growth restriction, preeclampsia) necessitating early delivery [2].

PROM described as the rupture of amniotic membranes before labor begins [5], can result in preterm labor, if onset is before 37 weeks of pregnancy, referred as ‘preterm premature rupture of membranes (PPROM)’. PROM constitutes about 8 to 10 percent of all PTB-pregnancies [5]. Membrane rupture results from a number of factors that ultimately lead to accelerated membrane weakening, with increase in cytokines, an imbalance in the interaction between matrix metalloproteinases and its tissue inhibitors, increased activity of collagenase and protease, and other factors which can cause increased intrauterine pressure [6, 7].

Pakistan sees 748,100 PTB cases annually, ranking fourth after India, China, and Nigeria, with an estimated high incidence of PTB of up to 16.5% [8].

The metabolic proteins (pathway) leading to PTB are overlapping between multiple etiologies; thus, understanding PTB initiators and effectors is difficult, preventing the discovery of biomarkers as usual predictors for this state [9]. Their early identification may result in the initiation of risk-specific management/treatment for high-risk women, and these risk factors may provide molecular insights into a better understanding of the mechanisms leading to PTB.

Complement factors B, H and the coagulation factors IX and IX ab have been identified as the highest-ranking proteins with distinguishing cases of PTB [10]. Similarly, an increased expression of the Bromodomain and Extra-Terminal motif (BET) proteins in the uterus and fetal membranes during labor/infection is seen. Lim and others reported BET proteins as novel therapeutic targets for reducing inflammation associated with spontaneous PTB [11]. A case-control has reported the higher concentrations of CRP (C-reactive protein), and Complement C3 levels in the first trimester that were associated with increased risk of PTB [12]. Recently, a study is performed that focused on the early identification of asymptomatic women at mid-gestation with the aid of novel protein biomarkers as preventive management to reduce the global maternal mortality burden in women with spontaneous PTB [13]. Recently, a research finding shared that placental STOX1 (STORKHEAD-BOX PROTEIN 1) transcription factor mutation (Y153H, C allele) is a contributing factor in PTB and placental preeclampsia due to defects in early utero-placental development [14].

Therefore, the research objective was to search the genes for the most potent PTB protein/biomarkers as well as to investigate the occurrence of region-specific genetic variations, their impact on proteins’ stability, evolutionary conservativeness, and changes in properties of protein associated with PTB. Moreover, to identify potential therapeutic targets with their corresponding protein cavities and their binding interactions with intervening bioactive compounds was also explored to manage the challenges of PTB.

## Materials and methods

### Gene and protein data retrieval

**Fig 1:**
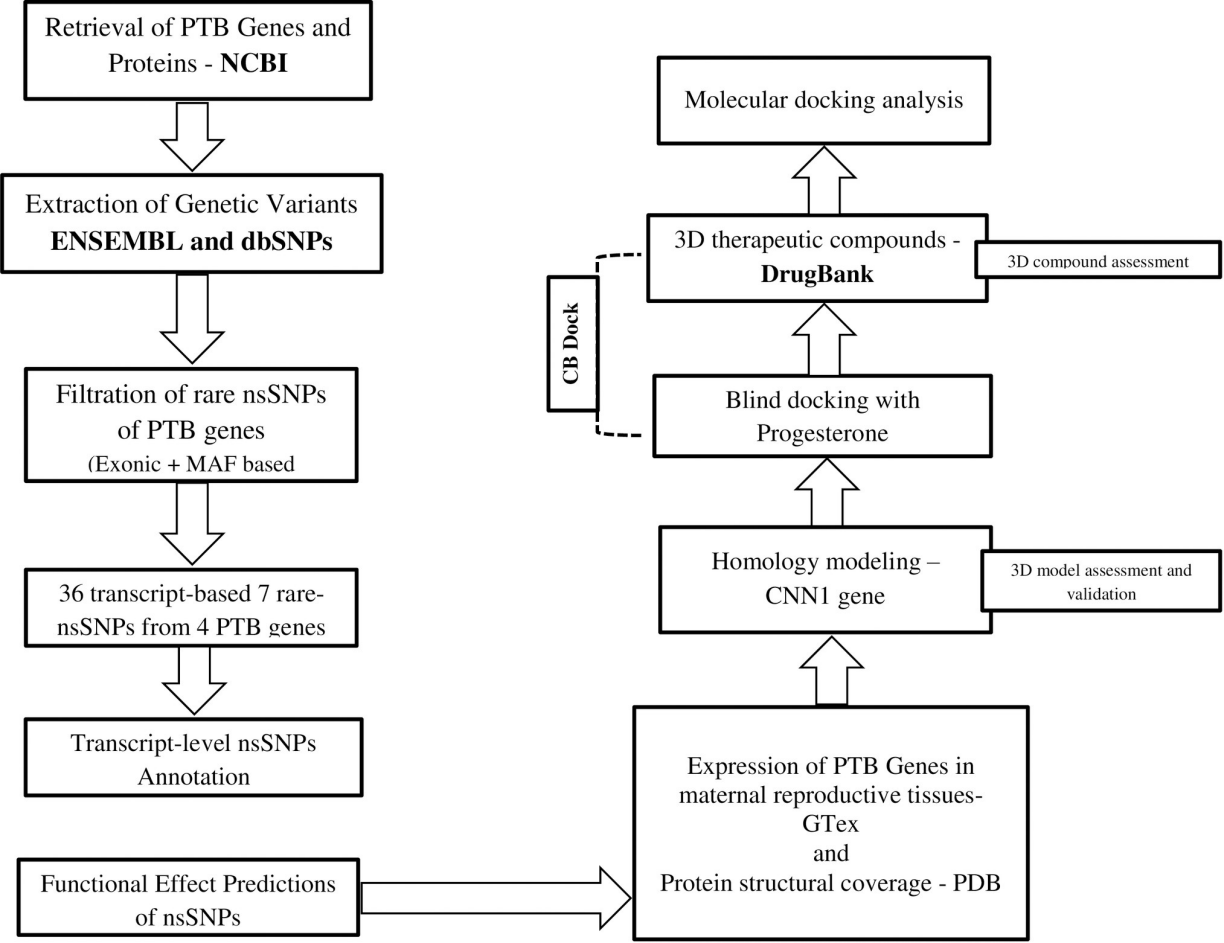
Schematic representation of workflow.

Most potent PTB-related genes were mined from the NCBI (the National Center for Biotechnology Information) databases (https://www.ncbi.nlm.nih.gov/genes). The search strategy included the key terms and synonyms, adapted to retrieve PTB-related genes and proteins, were *“preterm birth OR preterm labor OR preterm delivery”*. All the relevant genes along with their corresponding proteins were extracted from the period of 1^st^ January 2009 till 30^th^ June 2021. Overall, 55 different proteins from 20 different genes were found (various isoforms were also inclusive). All required genomic information, such as, genes names, aliases, HGNC (HUGO Gene Nomenclature Committee) Name/ID, NCBI accession number, ENSEMBL genes ID, genomic coordinates, gene type, transcripts counts, exon counts, genetic variation counts, exonic variation counts, filtered exonic variants, protein name with amino acids counts, UniProtKB Identifiers, reference sequence status, and gene expression were retrieved from NCBI https://www.ncbi.nlm.nih.gov/gene; ENSEMBL http://asia.ensembl.org/Homo_sapiens/Gene/Summary; HGNC (HUGO Gene Nomenclature Committee) https://www.genenames.org/; and GeneCards (https://www.genecards.org/) (for the workflow, refer to Fig 1).

### Extraction of non-synonymous single nucleotide polymorphisms (nsSNPs) from genes

Variations of each gene were extracted from Variant Effect Predictor (VEP) module [15] of ENSEMBL (https://asia.ensembl.org/index.html) database (Human Genome Assembly, GRCh38.p13 build 38), further validated by dbSNP (https://www.ncbi.nlm.nih.gov/snp/). VEP helps in the determination of downstream functional effects of variants (SNPs, deletions, insertions, structural variants or SNVs) on genes, transcripts, and protein sequence, as well as for regulatory regions. The relevant information of all SNPs included SNP ID, chromosome loci, nucleic acid changes, amino acid changes and reported transcript sequence ID. All SNPs were retrieved from ENSEMBL database, followed by the extraction of exonic variants by applying filter in the VEP module of ENSEMBL. The extracted data was downloaded as default comma-separated values (CSV) excel file.

### Selecting rare nsSNPs by population frequency distribution and downstream functional effects

1000 genome project database (1KGD) [16] was used to select rare nsSNPs based on minor allele frequency (MAF≤0.01 or 1%). Further filtering was performed by observing downstream functional impacts of rare nsSNPs by following *in-silico* algorithms:

#### Storing Intolerant from Tolerant (SIFT)

SIFT (https://sift.bii.a-star.edu.sg/) is used to distinguish pathogenic mutations having significant involvement with disease from neutral polymorphisms. In simple way, SIFT predictions helps to analyze the amino acid substitution effects on the function of protein [17]. SIFT score represents a normalized probability value of observing the new amino acid at that position with the range from 0 to 1. A value between 0 and 0.05 is predicted to affect protein functions.

#### Polymorphism Phenotyping (PolyPhen2)

The functional impact of substitution of an amino acid on collective proteins function and its stability was scored through PolyPhen2 (Polymorphism Phenotyping, http://genetics.bwh.harvard.edu/pph2/dbsearch.shtml). The prediction (score) output is categorized as ‘*most likely damaging*’, ‘*may be damaging*’, and ‘*benign’* with specificity and sensitivity values for a mutation [18] and was used as for gene annotation.

#### Combined Annotation Dependent Depletion (CADD) score

CADD is preferably used to filter the deleteriousness of SNVs as insertion/deletion variants in various genomic dataset of humans. CADD score with 20 or greater was chosen to select nsSNPs because this scale strongly correlates with allelic diversity, variant pathogenicity and experimentally measured regulatory effects within individual genome sequences [19].

#### The Rare Exome Variant Ensemble Learner (REVEL)

To assess the potential pathogenicity of SNV, REVEL is used to assess the pathogenicity of missense variants. This tool is not used as a descriptive prediction method but for ease, it displays scores greater ‘0.5’, as ’likely disease causing’ and display scores lower to ‘0.5’ as ’likely benign’. It was estimated that 75.4% of disease mutations but only 10.9% of neutral variants have a score above 0.5 [20].

#### MetaLR

This logistic regression-based tool is used to categorized SNV as ’damaging’ or ’tolerated’. In this case, a score between 0 and 1 is also provided and variants with higher scores are more likely to be deleterious [21].

#### Mutation assessor class

This tool predicts the functional impact of amino acid substitutions. The prediction is classified as ‘high’ that represented any variants with more likely to be deleterious in genomic datasets [22].

### Identification of regional nsSNPs by allele frequency aggregator (ALFA) of South Asian populations

Allele Frequency Aggregator (ALFA) is one of the largest and most comprehensive aggregated variant datasets of allele frequency with open access available from the NCBI database of Genotypes and Phenotypes (dbGaP) [23, 24]. ALFA computes the allele frequency for variant in dbGaP across approved un-restricted studies and provides the data as open access through dbSNP [25]. With the help of ALFA, the total sample size with reference to alternate allele of filtered/identified variants both globally and in South Asian populations were identified and listed.

### Transcript level analysis of the functional impacts of nsSNPs using different *in-silico* algorithms

All exonic missense SNPs, retrieved and analyzed through earlier mentioned strategies were further investigated by the transcript sequence-based computational analysis through various Machine Learning (ML) algorithms, to further scrutinized the nsSNPs as whether pathogenic or not. These *in silico* approaches include:

#### PANTHER-PSEP

PANTHER-PSEP was used to calculate the score of position-specific evolutionary preservations through online classification system, such as, http://www.pantherdb.org/tools/csnpScore.do. It measures the length of time (in millions of years ‘my’), a position in a current protein has been preserved by tracing back to its reconstructed direct ancestors. The longer a position has been preserved, the more likely that it will have a deleterious effect. The thresholds were: "probably damaging" (time > 450my, corresponding to a false positive rate of ~0.2 as tested on HumVar), "possibly damaging" (450my > time > 200my, corresponding to a false positive rate of ~0.4) and "probably benign" (time < 200my) [26].

#### PhD-SNP

PhD-SNP is a pathogenicity-predicting tool based on support vector machines through web server (https://snps.biofold.org/phd-snp/phd-snp.html). The Swiss-Prot database was used for training and the prediction was used with 20-fold cross validation [27].

#### PROVEAN

PROVEAN is sequence homology-based tools which have independently developed algorithms through web server available on http://provean.jcvi.org/index.php. The cutoff score of PROVEAN is -2.5 and mutations with scores over -2.5 are predicted to be pathogenic [28].

#### SNPs&GO

SNPs&GO from a protein sequence predict whether a variation is disease related or not by exploiting the corresponding protein functional annotation available on https://snps.biofold.org/snps-and-go/snps-and-go.html. SNPs&GO collects in unique framework information derived from protein sequence, evolutionary information, and function as encoded in the Gene Ontology (GO) terms, and outperforms other available predictive methods. SNPs&GO includes information extracted from the protein 3D structure (SNPs&GO3d) with respect to sequence-based method [29].

#### SNAP2

SNAP2 is a neural network-based classifier. SNAP2 predicts the impact (effect) of single amino acid substitutions on protein function (https://rostlab.org/services/snap2web/) that distinguishes between effect and neutral variants/nsSNPs by taking a variety of sequence and variant features into account. A high score (>50 indicates strong signal for effect), white indicates weak signals (-50<score<50), and a low score (score<-50, strong signal for neutral / no effect [30].

#### PMut

PMut (http://mmb.irbbarcelona.org/PMut/) is a web-based tool for the annotation of pathological variants on proteins, was trained and tested by the manually created database SwissVar (October 2016 release), which includes 27,203 harmful and 38,078 benign mutations for 12,141 proteins. The prediction scores of PMut are from 0 to 1, and the cutoff value is set to 0.5 (neutral, 0 to 0.5; pathological, 0.5 to 1) [31].

#### MutPred2

A web-server tool developed to categorize and predict amino acid substitutions on functional protein as pathogenic or benign in human, available through http://mutpred.mutdb.org/. The scores range from 0.5 to 1.0 are classify as ‘pathogenic mutation’ whereas, values less than 0.5 are ‘benign’ [32].

### *In-silico* supervised learning-based approaches to characterize nsSNPs impact on protein stability and evolutionary conservation associated with structural properties of proteins

#### MUpro

MUpro is a set of ML programs to predict how single-site amino acid mutation affects protein stability (http://mupro.proteomics.ics.uci.edu/). It was developed on the basis of two machine learning methods *i*.*e*., Support Vector Machines (SVM) and Neural Networks (NN). The advantage of this *in-silico* algorithm is that they do not require tertiary structures to predict protein stability changes [33].

#### I-Mutant

I-Mutant v.3.0 is a predictor of protein stability changes upon mutations. I-Mutant v.3.0 is an SVM-based tool for the automatic prediction of protein stability changes upon single point mutations at pH 7 and temperature equal to 25°C. The predictions were performed from the protein sequence and can be used both as a classifier for predicting the sign of the protein stability change upon mutation and as a regression estimator for predicting the related DeltaDeltaG (ΔΔG) values (http://gpcr2.biocomp.unibo.it/~emidio/I-Mutant3.0/I-MutantSuite_Help.html). Through I-Mutant predictions, the ternary classificatory has an extra class, namely Neutral, that refers to small ΔΔG values changes upon (WT/New) single point protein mutations. The range of ΔΔG values for a Neutral mutation classification is: -0.5 = <DDG = <0.5, while changes <-0.5 are classified as Large Decrease and changes >0.5 are classified as Large Increase [34].

#### iPTREE-STAB

iPTREE-STAB/iStable v.2.0 is an interpretable decision tree-based method for discriminating the stability of proteins stabilizing or destabilizing (predicting their stability changes, ΔΔG) upon single amino acid substitutions from amino acid sequence. The discrimination and predictions are mainly based on decision tree coupled with adaptive boosting algorithm, and classification and regression tree, respectively, using three neighboring residues of the mutant site along N- and C-terminals (http://ncblab.nchu.edu.tw/iStable2/seqsubmit.html). This tool also computes the secondary structural information and surface accessibility of protein sequence with mutation [35, 36].

#### INPS-MD

INPS-MD (Impact of Non-synonymous mutations on Protein Stability-Multi Dimension, (https://inpsmd.biocomp.unibo.it) is a method used to predict stability of protein variants from sequences and structures. The INPS-MD predictor using sequences is based on a simplified support vector (SVR) as implemented by the *libsvm* package, which was only tested by linear and radial basis function (RBF) kernels. INPS-MD predictions can be interpreted to identify stabilizing (ΔΔG>0) and destabilizing (ΔΔG<0) variations [37].

#### ConSurf

ConSurf software (http://consurf.tau.ac.il) was used to analyze the evolutionary conservation of amino acids by calculating the conservation score through unique algorithm. The algorithm calculates normalized conservative score using Bayesian method for calculating rates with a confidence interval to each of the inferred evolutionary conservation scores. The amino acids with scores between 7 and 9 were evolutionary conservative amino acids [38].

#### SOPMA

The SOPMA—a neural network method (PHD), jointly predict the secondary structure (α helix, β turn and coil) of amino acid residues. This software (https://npsa-prabi.ibcp.fr/cgi-bin/npsa_automat.pl?page=npsa_sopma.html) used five independent algorithms to compute the secondary structural details of proteins [39].

#### HOPE

The HOPE software (Have (y) Our Protein Explained, http://www.cmbi.ru.nl/hope/input/) was used to predict the effect of amino acid mutation/SNPs on physical and chemical properties, hydrophobicity, spatial structure, and function of proteins [40].

### Exploring protein structural coverage expressed in female reproductive tissues

The Genotype-Tissue Expression (GTEx) database cataloged a large number of tissue-specific genotypes and shared regulatory expression quantitative trait loci (eQTL) variants. We used GTEx to identify the best expressed gene in the female reproductive tissues (bladder, cervix–ectocervix, endocerivx, fallopian tubes, ovaries, uterus, and vagina) that could impact PTB status at protein level [41]. After investigating the concerned genes/proteins with identified tissue-specific regulatory expression in female reproductive tissues, the Protein Data Bank (PDB) structural coverage details from previous reported studies were investigated from PDB [42].

### Homology modeling and 3D structural assessment of highly expressed PTB protein

Protein Homology/analogY Recognition Engine V 2.0 (Phyre2) tool was used for homology modeling of concerned PTB protein [43]. Following two attributes in protein selection for homology modeling were also kept considered for inclusion:

1. Protein highly expressed with a large number of tissue-specific and shared regulatory variants in female reproductive tissues.
2. Protein with highest structural coverage information (retrieved through PDB) among others (found with rare coding variants).

After following the above-mentioned inclusion criteria, CNN1 was selected for 3D homology modeling. The target sequence of Calponin1 (CNN1 Isoform 2) protein was searched from UniProt database (Entry No. P51911-2). PHYRE2 tool was used for homology modeling that consisted of sub-algorithmic stages:

***Stage 1—Gathering Homologous Sequences*:** CNN1 isoform 2 sequence was scanned against the specially curated NR20 (No of sequences with >20% mutual sequence identity) protein sequence database with HHblits [44]. The resulting Multiple Sequence Alignment (MSA) was used to predict the secondary structure with PSI-blast based secondary structure PREDiction (PSIPRED) [45], and both the alignment and secondary structure prediction combined into a query Hidden Markov Model (HMM).

***Stage 2—Fold Library Scanning*:** The models were scanned against a database of HMMs [46] of proteins of known structure. The top-scoring alignments from this search were used to construct crude backbone-only models.

***Stage 3*—*Loop Modeling*:** Indels in these models were corrected by loop modeling.

***Stage 4*—Side-chain Placement:** Amino acid side chains were added to generate the final PHYRE2 models.

The best homology models were selected based on the top-ranked modeled structures (according to similar template pattern). The alignment description of templates obtained through manual protein-blast comparison with the highest percent identity at 100% confidence was generated by Phyre2 tool. The quality assessment of stereochemical properties of 3D homology models were carried out by PROCHECK [47]. Ramachandran plot and residual properties of constructed 3D model along with the dihedral angles of φ against ψ of all possible conformations of amino acids in protein structure had also been studied in Ramachandran plot [48]. The validation of 3D protein models were also performed by SAVES (Structure Validation Server: https://saves.mbi.ucla.edu/) web server [49] to know the probable structural errors and *z*-score of the chosen model. SMART-EMBL tool (http://smart.embl-heidelberg.de/) was used to compute the confidently predicted domains, repeats, motifs and low complexity region (LCR) in protein. The SuperPose webserver (http://superpose.wishartlab.com/) was used to calculate both sequence alignment between template and 3D homology model through structure superposition using modified quaternion eigenvalue approach to generate RMSD statistics of the superimposed molecules [50].

### Validation of binding cavities by consensus mode

To detect protein binding cavities, cavity-detection guided Blind Docking approach was used to explore the binding cavities of homology models. CB-Dock is an automatic protein-ligand docking approach which identifies the binding cavities/sites. CB dock compared cavities and ranked through a method called ‘CurPocket’ with state-of-the-art protein–ligand binding site prediction methods using the benchmark set of COACH [51], as prediction methods. CB dock also calculates the center and size of docking box of putative cavity as a key parameter of process with novel curvature-based cavity detection approach. This method was carefully optimized and achieved ~70% success rate for the top-ranking poses whose root mean square deviation (RMSD) were within 2 Å from the X-ray pose [52]. Progesterone (as various synthetic derivate are commonly used to manage PTB) was used as ligand molecule, downloaded from the PUBCHEM compound library (PubChem CID 5994).

### Selection of drug molecules involved in PTB management

Drug used in PTB management were explored from the publicly accessible DrugBank database having comprehensive information about drugs and drug targets [53]. The search strategy included the key terms and synonyms, adapted to retrieve PTB-related drugs and therapeutics were *“preterm birth OR preterm labor OR preterm delivery”*. The visual inspection of all compounds was performed to ensure the arrangements of respective constituting atoms as per 3D molecular structures. Furthermore, stable molecular conformations with arrangement in space was carried out by the energy minimization of 3D structures of PTB therapeutic compounds. According to *‘drug type’* category, only small molecules were filtered by Lipinski’s rule of five [54]. After analysis, only those PTB drugs were selected which had PASS all five physicochemical properties of Lipinski’s rule, as any single violation were treated as FAIL.

### *In-silico* molecular mechanics and energetic estimations of PTB drugs interacted with CNN1

AutoDock Vina software (version 4.2) was used for protein-drug analysis and to estimate the molecular interactions of PTB proteins with selected drugs (ligand) molecules [55]. The best obtained cavity binding pattern (homology model with progesterone) of CB-Dock with lowest free energy and RMSD value were selected, and molecular interacting targets were further used as prominent residues in this cavity-guided AutoDock approach. Polar hydrogens were added, and partial charges were assigned to the standard residue using Gasteiger partial charge, which assumes that all hydrogen atoms were represented explicitly. The most favorable binding interactions were estimated through lowest predicted free energy of binding with best molecular docking simulation pose.

The interactions of the docked complexes and 2D ligand-protein interaction plots were drawn with the help of Discovery Studio 2017 R2 Client (v17.2.0.16349) available at https://discover.3ds.com/discovery-studio-visualizer-download.

### *In-silico* pharmacokinetics predictions of the screened PTB drugs

The ADMET (Absorption, Distribution, Metabolism, Excretion, and Toxicity) approach was used for *in-silico* pharmacokinetic predictions of the selected PTB drugs. ADMET lab platform (http://admet.scbdd.com/home/index/) [56] was used to access the ADMET properties. The assessment was carried out for each physiochemical property by submitting a SMILE format of the individual compound.

## Results

### PTB-related genes and proteins

Overall, 55 proteins (isoforms inclusive) of 20 genes were found with PTB-related search query from NCBI duration from 1^st^ January, 2009 to 30^th^ June, 2021) (Table 1). Out of 20 genes, 18 genes have had reviewed reference sequence status while the remaining two have validated status (SKA2 and CNN1). 9 genes were located on the reverse strands and 11 genes were located on the forward strand. The significant gene expression in endometrium and other female reproductive tissues were exhibited by 9 genes, including PAEP, SERPINH1, PLAG1, CNN1, IL6, ADAMTS4, SIGLEC6, PBX1, and KLRC1. PLAG1 gene has the highest transcription counts of 28. The total exon counts were also extracted, which indicated that COL24A1 gene has the highest numbers of exons (66) while IQGAP and SLIT2 have 41 and 40 exons, respectively. The highest numbers of isoforms were found in PLAG1, i.e., 20 isoforms (Table 1).

**Table 1:** List of PTB-related genes and proteins extracted from the NCBI database with retrieval of genomic information.

S. No.	Gene Official Full Name	NCBI Acc. No.	Chromosomal Location	Transcript Counts/Exon Counts*	Genetic Variation Counts	Exonic Variation Counts	Protein Name	Amino Acid Counts	UniProtKB Identifier	Gene Expression
**1**	adenylate cyclase 5—ADCY5	NP_001365188, XP_005247134	3:123,282,296–123,449,758 reverse strand	10/30	42,644	6,600	adenylate cyclase type 5 isoform 3	1,286	O95622	Heart, brain and 17 other tissues
**2**	adenylate cyclase 5—ADCY5	NP_001186571	3:123,282,296–123,449,758 reverse strand	10/30	42,644	6,600	adenylate cyclase type 5 isoform 2	911	O95622	Heart, brain and 17 other tissues
**3**	slit guidance ligand 2—SLIT2	NP_001276064, XP_005248269	4:20,251,905–20,620,561 forward strand	12/40	92,881	13,423	slit homolog 2 protein isoform 2 precursor	1,525	O94813	Lung, adrenal and 211 other tissues
**4**	slit guidance ligand 2—SLIT2	NP_001276065, XP_005248270	4:20,251,905–20,620,561 forward strand	12/40	92,881	13,423	slit homolog 2 protein isoform 3 precursor	1,521	O94813	Lung, adrenal and 211 other tissues
**5**	roundabout guidance receptor 1—ROBO1	NP_001139317	3:78,597,239–79,767,998 reverse strand	13/35	278,967	16,467	roundabout homolog 1 isoform d	1,551	Q9Y6N7	Ubiquitous expression in brain, skin, and 23 other tissues
**6**	progestagen associated endometrial protein—PAEP^	NP_001018058, XP_005263462	9:135,561,756–135,566,955 forward strand	9/7	14,101	2,294	glycodelin isoform 2 precursor	158	P09466	Restricted expression toward endometrium
**7**	interleukin 1 receptor antagonist—IL1RN	NP_001366289, XP_005263718	2:113,107,214–113,134,016 forward strand	9/11	28,215	1,442	interleukin—1 receptor antagonist protein isoform 4	143	P18510	Biased expression in esophagus, bone marrow, and 2 other tissues
**8**	interleukin 1 receptor antagonist—IL1RN	NP_001305843, XP_006712560	2:113,107,214–113,134,016 forward strand	9/11	28,215	1,442	interleukin—1 receptor antagonist protein isoform 4	143	P18510	Biased expression in esophagus, bone marrow, and 2 other tissues
**9**	pre T cell antigen receptor alpha—PTCRA	NP_001230098	6:42,915,989–42,925,838 forward strand	4/6	12,538	1,659	pre T—cell antigen receptor alpha isoform 3 precursor	256	Q6ISU1	Biased expression in spleen, lymph node (RPKM 0.8) and 5 other tissues
**10**	pre T cell antigen receptor alpha—PTCRA	NP_001230097	6:42,915,989–42,925,838 forward strand	4/6	12,538	1,659	pre T—cell antigen receptor alpha isoform 3 precursor	296	Q6ISU1	Biased expression in spleen, lymph node and 5 other tissues
**11**	pre T cell antigen receptor alpha—PTCRA	NP_001230099	6:42,915,989–42,925,838 forward strand	4/6	12,538	1,659	pre T—cell antigen receptor alpha isoform 3 precursor	174	Q6ISU1	Biased expression in spleen, lymph node and 5 other tissues
**12**	spindle and kinetochore associated complex subunit 2—SKA2	NP_001317328, XP_011523047	17:59,109,857–59,155,260 reverse strand	10/4	12,173	1,059	spindle and kinetochore—associated protein 2 isoform 3	109	Q8WVK7	Ubiquitous expression in brain, thyroid and 25 other tissues
**13**	serpin family H member 1—SERPINH1^	NP_001193943	11:75,562,056–75,572,783 forward strand	16/8	29,620	7,471	serpin H1 precursor	418	P50454	Broad expression in placenta, endometrium and 21 other tissues
**14**	interleukin 4—IL4	NP_001341919	5:132,673,986–132,682,678 forward strand	4/5	8363	630	interleukin—4 isoform 3 precursor	136	P05112	Low expression observed in reference dataset
**15**	PLAG1 like zinc finger 1—PLAGL1^	NP_001304091	6:143,940,300–144,064,599 reverse strand	28/12	28,906	9,151	zinc finger protein PLAGL1 isoform 2	463	Q9UM63	Broad expression in placenta, skin and 16 other tissues
**16**	PLAG1 like zinc finger 1—PLAGL1^	NP_001304090	6:143,940,300–144,064,599 reverse strand	28/12	28,906	9,151	zinc finger protein PLAGL1 isoform 2	463	Q9UM63	Broad expression in placenta, skin and 16 other tissues
**17**	PLAG1 like zinc finger 1—PLAGL1^	NP_001304089	6:143,940,300–144,064,599 reverse strand	28/12	28,906	9,151	zinc finger protein PLAGL1 isoform 1	411	Q9UM63	Broad expression in placenta, skin and 16 other tissues
**18**	PLAG1 like zinc finger 1—PLAGL1^	NP_001304088	6:143,940,300–144,064,599 reverse strand	28/12	28,906	9,151	zinc finger protein PLAGL1 isoform 2	463	Q9UM63	Broad expression in placenta, skin and 16 other tissues
**19**	PLAG1 like zinc finger 1—PLAGL1^	NP_001304087	6:143,940,300–144,064,599 reverse strand	28/12	28,906	9,151	zinc finger protein PLAGL1 isoform 1	411	Q9UM63	Broad expression in placenta, skin and 16 other tissues
**20**	PLAG1 like zinc finger 1—PLAGL1^	NP_001304086	6:143,940,300–144,064,599 reverse strand	28/12	28,906	9,151	zinc finger protein PLAGL1 isoform 2	463	Q9UM63	Broad expression in placenta, skin and 16 other tissues
**21**	PLAG1 like zinc finger 1—PLAGL1^	NP_001304085	6:143,940,300–144,064,599 reverse strand	28/12	28,906	9,151	zinc finger protein PLAGL1 isoform 2	463	Q9UM63	Broad expression in placenta, skin and 16 other tissues
**22**	PLAG1 like zinc finger 1—PLAGL1^	NP_001275968, XP_005267083	6:143,940,300–144,064,599 reverse strand	28/12	28,906	9,151	zinc finger protein PLAGL1 isoform 1	411	Q9UM63	Broad expression in placenta, skin and 16 other tissues
**23**	PLAG1 like zinc finger 1—PLAGL1^	NP_001275967	6:143,940,300–144,064,599 reverse strand	28/12	28,906	9,151	zinc finger protein PLAGL1 isoform 1	411	Q9UM63	Broad expression in placenta, skin and 16 other tissues
**24**	PLAG1 like zinc finger 1—PLAGL1^	NP_001275978, XP_005267079	6:143,940,300–144,064,599 reverse strand	28/12	28,906	9,151	zinc finger protein PLAGL1 isoform 2	463	Q9UM63	Broad expression in placenta, skin and 16 other tissues
**25**	PLAG1 like zinc finger 1—PLAGL1^	NP_001275974	6:143,940,300–144,064,599 reverse strand	28/12	28,906	9,151	zinc finger protein PLAGL1 isoform 2	463	Q9UM63	Broad expression in placenta, skin and 16 other tissues
**26**	PLAG1 like zinc finger 1—PLAGL1^	NP_001275972	6:143,940,300–144,064,599 reverse strand	28/12	28,906	9,151	zinc finger protein PLAGL1 isoform 2	463	Q9UM63	Broad expression in placenta, skin and 16 other tissues
**27**	PLAG1 like zinc finger 1—PLAGL1^	NP_001275977, XP_005267078	6:143,940,300–144,064,599 reverse strand	28/12	28,906	9,151	zinc finger protein PLAGL1 isoform 2	463	Q9UM63	Broad expression in placenta, skin and 16 other tissues
**28**	PLAG1 like zinc finger 1—PLAGL1^	NP_001275975	6:143,940,300–144,064,599 reverse strand	28/12	28,906	9,151	zinc finger protein PLAGL1 isoform 2	463	Q9UM63	Broad expression in placenta, skin and 16 other tissues
**29**	PLAG1 like zinc finger 1—PLAGL1^	NP_001275966	6:143,940,300–144,064,599 reverse strand	28/12	28,906	9,151	zinc finger protein PLAGL1 isoform 1	411	Q9UM63	Broad expression in placenta, skin and 16 other tissues
**30**	PLAG1 like zinc finger 1—PLAGL1^	NP_001275969, XP_005267086	6:143,940,300–144,064,599 reverse strand	28/12	28,906	9,151	zinc finger protein PLAGL1 isoform 1	411	Q9UM63	Broad expression in placenta, skin and 16 other tissues
**31**	PLAG1 like zinc finger 1—PLAGL1^	NP_001275976	6:143,940,300–144,064,599 reverse strand	28/12	28,906	9,151	zinc finger protein PLAGL1 isoform 2	463	Q9UM63	Broad expression in placenta, skin and 16 other tissues
**32**	PLAG1 like zinc finger 1—PLAGL1^	NP_001275970, XP_005267084	6:143,940,300–144,064,599 reverse strand	28/12	28,906	9,151	zinc finger protein PLAGL1 isoform 1	411	Q9UM63	Broad expression in placenta, skin and 16 other tissues
**33**	PLAG1 like zinc finger 1—PLAGL1^	NP_001275971	6:143,940,300–144,064,599 reverse strand	28/12	28,906	9,151	zinc finger protein PLAGL1 isoform 2	463	Q9UM63	Broad expression in placenta, skin and 16 other tissues
**34**	PLAG1 like zinc finger 1—PLAGL1^	NP_001275973	6:143,940,300–144,064,599 reverse strand	28/12	28,906	9,151	zinc finger protein PLAGL1 isoform 2	463	Q9UM63	Broad expression in placenta, skin and 16 other tissues
**35**	A—kinase anchoring protein 10—AKAP10	NP_001317081, XP_006721495	17:19,904,302–19,978,343 reverse strand	11/16	86,215	2,430	A—kinase anchor protein 10, mitochondrial isoform 2	604	O43572	Ubiquitous expression in thyroid, testis and 25 other tissues
**36**	calponin 1—CNN1^	NP_001295271, XP_011525993	19:11,538,767–11,550,323 forward strand	10/10	20,312	2,759	calponin—1 isoform 2	247	P51911	Biased expression in urinary bladder, endometrium and 11 other tissues
**37**	calponin 1—CNN1^	NP_001295270	19:11,538,767–11,550,323 forward strand	10/10	20,312	2,759	calponin—1 isoform 2	247	P51911	Biased expression in urinary bladder, endometrium and 11 other tissues
**38**	IQ motif containing GTPase activating protein 2—IQGAP2	NP_001272390, XP_005248473	5:76,403,285–76,708,132 forward strand	19/41	68,993	12,239	ras GTPase—activating—like protein IQGAP2 isoform 3	1,071	Q13576	Broad expression in liver, duodenum and 21 other tissues
**39**	IQ motif containing GTPase activating protein 2—IQGAP2	NP_001272389, XP_005248469	5:76,403,285–76,708,132 forward strand	19/41	68,993	12,239	ras GTPase—activating—like protein IQGAP2 isoform 2	1,525	Q13576	Broad expression in liver, duodenum and 21 other tissues
**40**	IQ motif containing GTPase activating protein 2—IQGAP2	NP_001272391, XP_005248472	5:76,403,285–76,708,132 forward strand	19/41	68,993	12,239	ras GTPase—activating—like protein IQGAP2 isoform 4	1,071	Q13576	Broad expression in liver, duodenum and 21 other tissues
**41**	collagen type XXIV alpha 1 chain—COL24A1	NP_001336884	1:85,729,233–86,156,943 reverse strand	5/66	98,890	5,150	collagen alpha—1(XXIV) chain isoform 2	1,014	Q17RW2	Low expression observed in reference dataset
**42**	interleukin 6—IL6^	NP_001358025	7:22,725,884–22,732,002 forward strand	9/6	11,090	2,040	interleukin—6 isoform 3	189	P05231	Broad expression in urinary bladder, gall bladder and 14 other tissues
**43**	interleukin 6—IL6^	NP_001305024, XP_011513693	7:22,725,884–22,732,002 forward strand	9/6	11,090	2,040	interleukin—6 isoform 2	136	P05231	Broad expression in urinary bladder, gall bladder and 14 other tissues
**44**	ADAM metallopeptidase with thrombospondin type 1 motif 4—ADAMTS4^	NP_001307265, XP_006711698	1:161,184,302–161,199,054 reverse strand	3/10	5757	1,895	A disintegrin and metalloproteinase with thrombospondin motifs 4 isoform 2 precursor	846	O75173	Broad expression in gall bladder, appendix and 19 other tissues
**45**	sialic acid binding Ig like lectin 6—SIGLEC6^	NP_001171020	19:51,517,819–51,531,856 reverse strand	9/11	26,337	4,404	sialic acid—binding Ig—like lectin 6 isoform 6 precursor	342	O43699	Biased expression in placenta and lung
**46**	sialic acid binding Ig like lectin 6—SIGLEC6^	NP_001171019	19:51,517,819–51,531,856 reverse strand	9/11	26,337	4,404	sialic acid—binding Ig—like lectin 6 isoform 5 precursor	389	O43699	Biased expression in placenta and lung
**47**	sialic acid binding Ig like lectin 6—SIGLEC6^	NP_001171018	19:51,517,819–51,531,856 reverse strand	9/11	26,337	4,404	sialic acid—binding Ig—like lectin 6 isoform 4	401	O43699	Biased expression in placenta and lung
**48**	PBX homeobox 1—PBX1^	NP_001340059	1:164,555,584–164,899,296 forward strand	22/11	79,096	4,272	pre—B—cell leukemia transcription factor 1 isoform 4	347	P40424	Ubiquitous expression in endometrium, gall bladder and 22 other tissues
**49**	PBX homeobox 1—PBX1^	NP_001340060, XP_016856885	1:164,555,584–164,899,296 forward strand	22/11	79,096	4,272	pre—B—cell leukemia transcription factor 1 isoform 2	347	P40424	Ubiquitous expression in endometrium, gall bladder and 22 other tissues
**50**	PBX homeobox 1—PBX1^	NP_001191892	1:164,555,584–164,899,296 forward strand	22/11	79,096	4,272	pre—B—cell leukemia transcription factor 1 isoform 3	420	P40424	Ubiquitous expression in endometrium, gall bladder and 22 other tissues
**51**	PBX homeobox 1—PBX1^	NP_001191890	1:164,555,584–164,899,296 forward strand	22/11	79,096	4,272	pre—B—cell leukemia transcription factor 1 isoform 2	347	P40424	Ubiquitous expression in endometrium, gall bladder and 22 other tissues
**52**	autophagy related 16 like 1—ATG16L1	NP_001350671, XP_005246139	2:233,210,051–233,295,674 forward strand	18/19	23,650	8,865	autophagy—related protein 16–1 isoform 6	624	Q676U5	Ubiquitous expression in bone marrow, testis and 25 other tissues
**53**	autophagy related 16 like 1—ATG16L1	NP_001177196	2:233,210,051–233,295,674 forward strand	18/19	23,650	8,865	autophagy—related protein 16–1 isoform 5	491	Q676U5	Ubiquitous expression in bone marrow, testis and 25 other tissues
**54**	autophagy related 16 like 1—ATG16L1	NP_001177195	2:233,210,051–233,295,674 forward strand	18/19	23,650	8,865	autophagy—related protein 16–1 isoform 4	523	Q676U5	Ubiquitous expression in bone marrow, testis and 25 other tissues
**55**	killer cell lectin like receptor C1—KLRC1^	NP_001291377, XP_005253417	12:10,442,264–10,454,685 reverse strand	7/9	13,767	1,977	NKG2—A/NKG2—B type II integral membrane protein isoform C	228	P26715	Broad expression in spleen, endometrium and 21 other tissues

*Exon count represents the largest transcript of the respective genes

^Significant expression in female reproductive tissues

### nsSNPs of PTB genes

**Fig 2:**
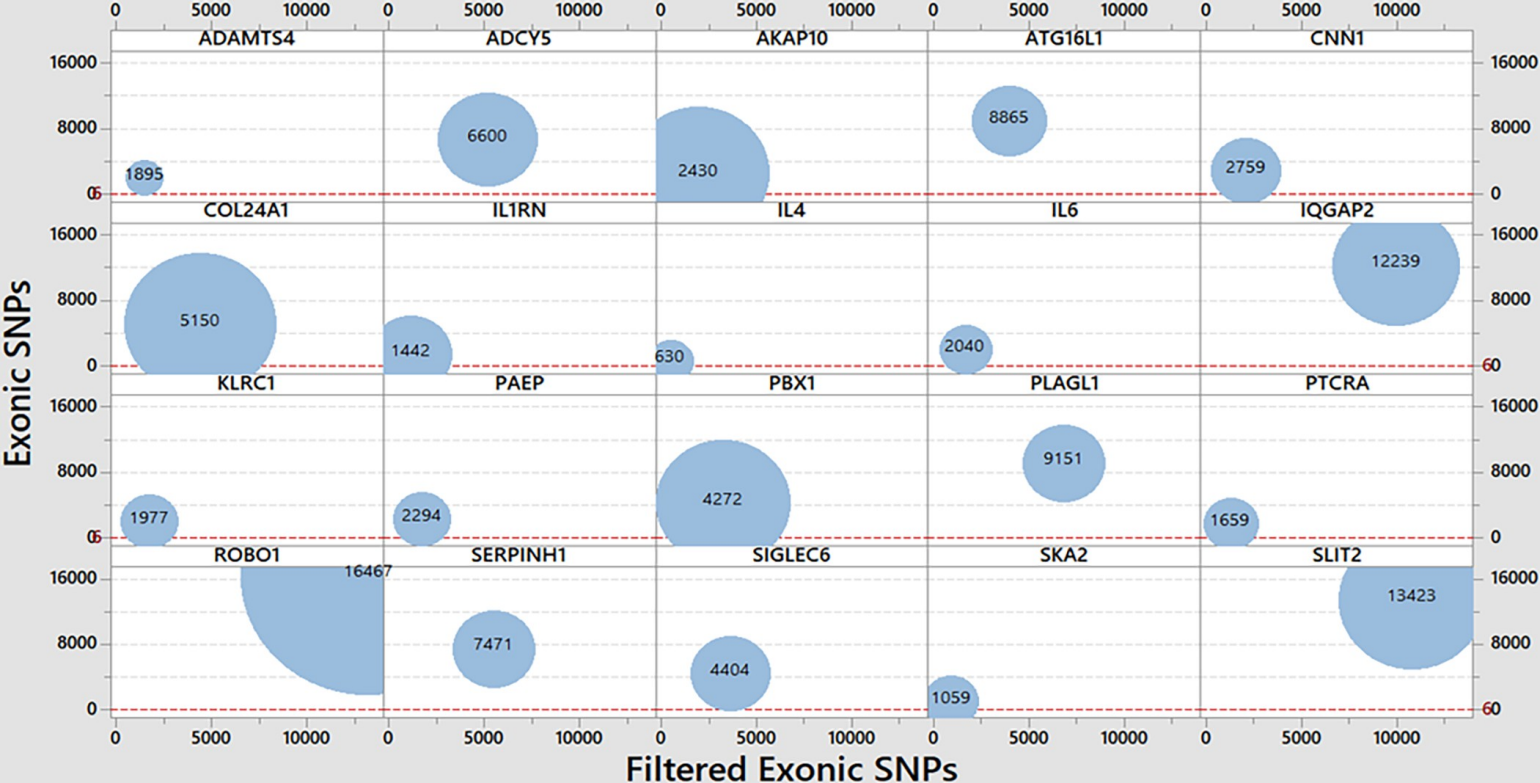
SNPs in the identified PTB-related genes. Bubble plot showing identified PTB-gene wise SNPs, exonic-SNPs and filtered exonic-SNPs. Each bubble size stands for the total count of SNPs.

Through ENSEMBL gene ID of each gene, the genetic variations were extracted. ROBO1 gene has the highest numbers of SNPs, i.e., 278,967, while ADAMTS4 has very least numbers of SNPs, i.e., 5,757 (Fig 2). Likewise, the highest exonic SNPs in the identified PTB-related genes were observed in ROBO1 genes, i.e., 16,467 while the lowest exonic SNPs were found in IL4, i.e., 630. The exonic SNPs were also filtered with the help of VEP module. Total numbers of nsSNPs were observed with much reduced in numbers as compared to the previous exonic counts in each gene. The nsSNPs were highest in ROBO1 gene (13,235) and IL4 has the lowest numbers of nsSNPs, i.e., 467 (Fig 2).

### Extraction of rare high risk nSNPs supported by transcript level computation of ALFA in South Asian population of PTB genes

**Fig 3:**
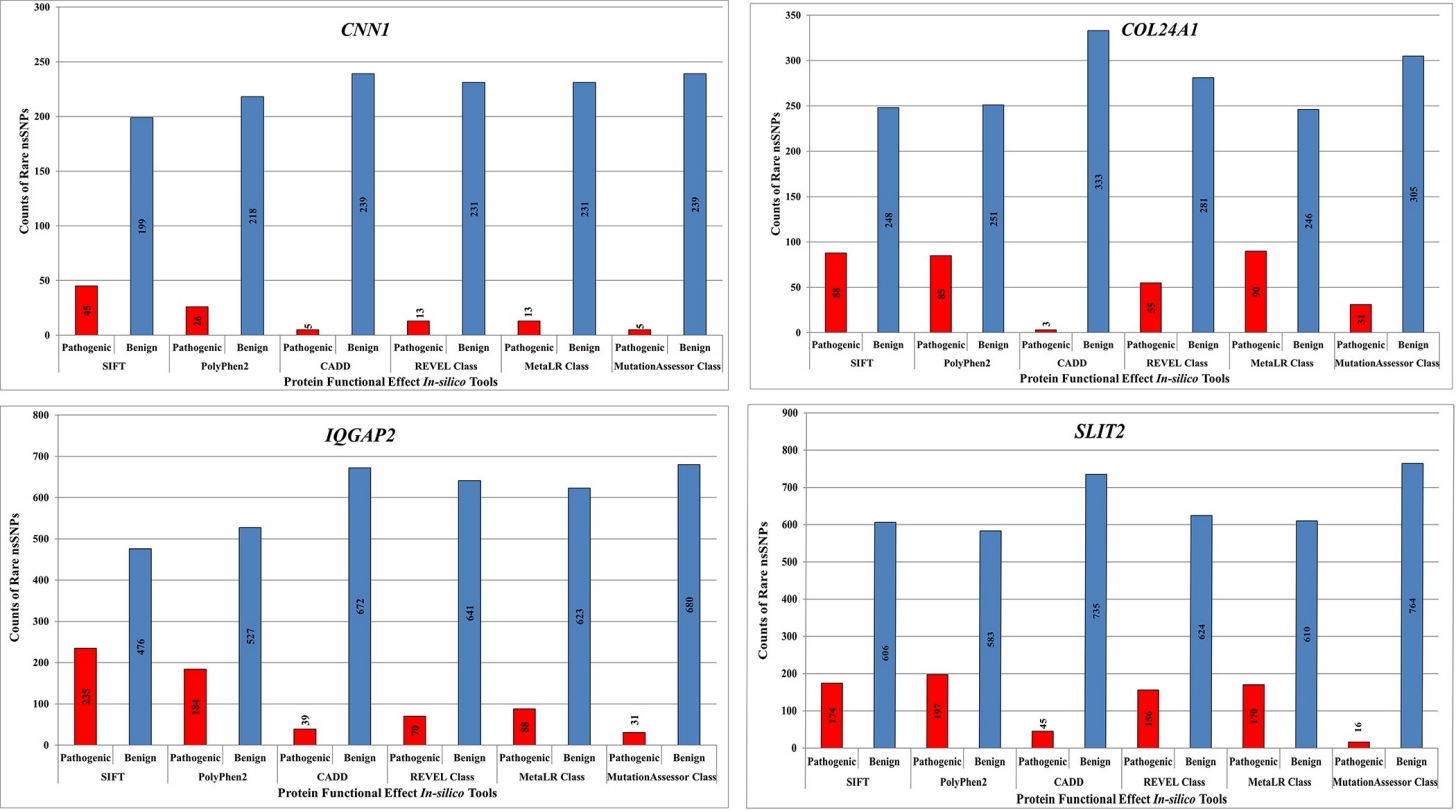
Downstream functional effect prediction of pathogenic SNPs based on various *in-silico* tools. A. *CNN1*, B. *COL24A1*, C. *IQGAP2*, and D. *SLIT2*.

**Fig 4:**
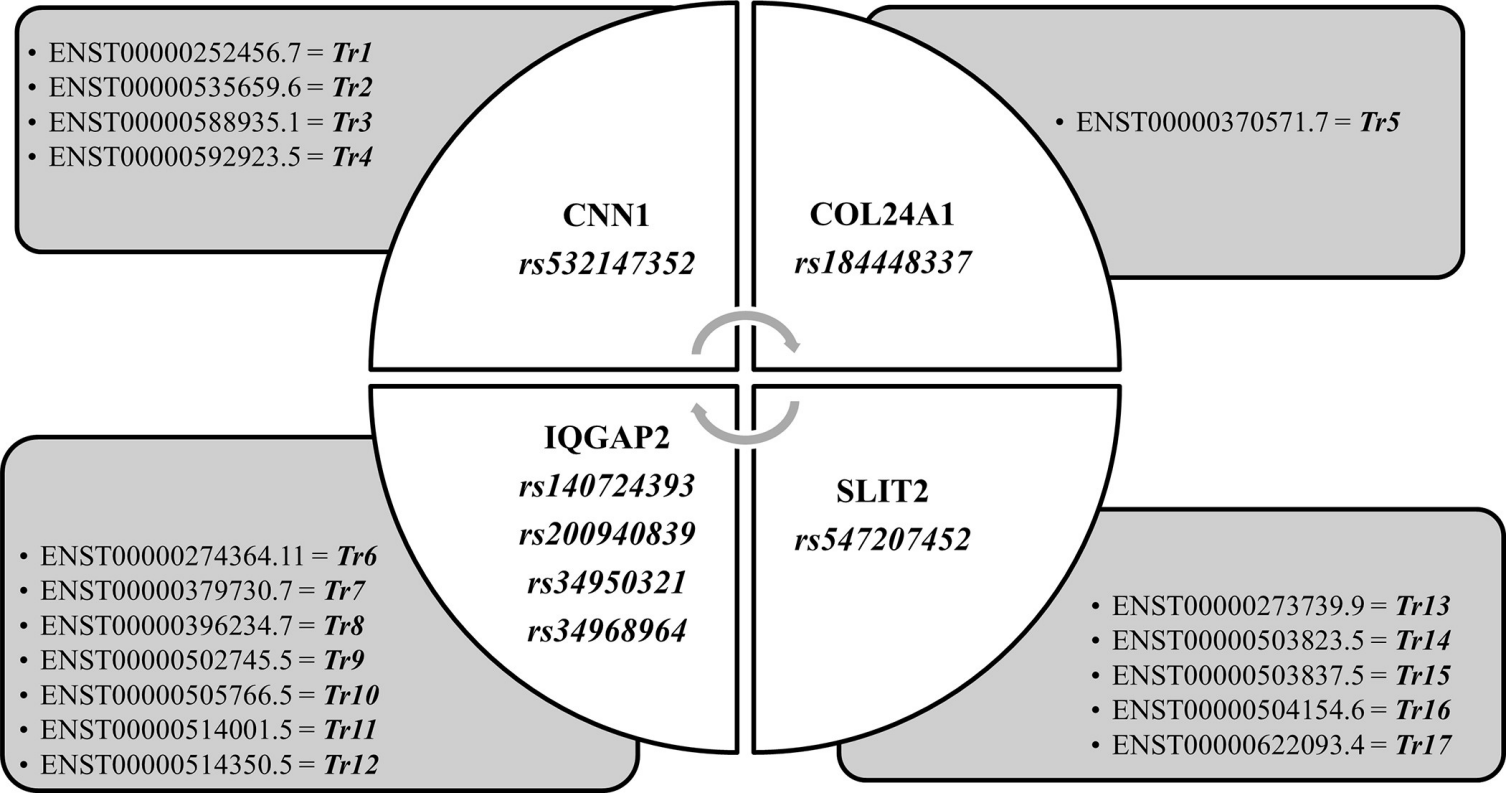
Distribution of rare nsSNPs based on transcript sequences. Filtered genes are shown based on pathogenic SNPs extracted through annotation strategies and their respective ENSEMBL transcripts versions (*Tr*).

All exonic variants of 20 PTB-related genes were further filtered based on MAF≤0.01 and downstream functional effects (Fig 3). Based on transcript sequences, the output kept only 4 genes having 36 rare coding variants, that is, CNN1 with 4, COL24A1 with 1, IQGAP2 with 26 and SLIT2 with 5 nsSNPs (Fig 4). The various gene annotation statuses of all 4 genes having deleterious nsSNPs were shown in Table 2. Fig 4 showed the distribution of nsSNPS of all 4 genes with their transcript sequences. To estimate the global impact of identified genomic variants, the computed ALFA frequencies of both global and in South Asian populations were identified. It was observed that ALFA frequencies were available only for two genes i.e., COL24A1 and IQGAP2 (S1 Table). The total count of rare nsSNPs identified in previous steps (36) was reduced to 27 (S1 Table). 1 deleterious variant was identified from COL24A1 gene, and 26 deleterious variants were from IQGAP2 which have computed ALFA (Global and South Asian) sample size with frequency details of reference and alternate alleles (S1 Table).

**Table 2:** Rare nsSNPs extracted on the basis of MAF and downstream functional effects of PTB-related genes.

Gene	rsID*	chr:Location	Alleles	Global MAF	Amino Acid Mutation	Evidence	Consequence Type	CADD	Transcript
CNN1	rs532147352	19:11549378	G/A	< 0.001	R186H	1000Genomes~ExAC~gnomAD	Missense Variant	32	ENST00000252456.7
CNN1	rs532147352	19:11549378	G/A	< 0.001	R136H	1000Genomes~ExAC~gnomAD	Missense Variant	32	ENST00000535659.6
CNN1	rs532147352	19:11549378	G/A	< 0.001	R42H	1000Genomes~ExAC~gnomAD	Missense Variant	32	ENST00000588935.1
CNN1	rs532147352	19:11549378	G/A	< 0.001	R136H	1000Genomes~ExAC~gnomAD	Missense Variant	32	ENST00000592923.5
COL24A1	rs184448337	1:85842393	C/T	< 0.001	G1155R	1000Genomes~ExAC~gnomAD	Missense Variant~Splice Region Variant	33	ENST00000370571.7
IQGAP2	rs140724393	5:76575751	A/G	< 0.001	Y147C	1000Genomes~ExAC~TOPMed~gnomAD	Missense Variant	31	ENST00000274364.11
IQGAP2	rs34968964	5:76665143	G/C	0.001	E883Q	1000Genomes~ESP~Phenotype_or_Disease~ExAC~TOPMed~gnomAD	Missense Variant	31	ENST00000274364.11
IQGAP2	rs34950321	5:76668682	C/T	0.005	T894I	1000Genomes~ESP~Phenotype_or_Disease~ExAC~TOPMed~gnomAD	Missense Variant~Splice Region Variant	32	ENST00000274364.11
IQGAP2	rs200940839	5:76673589	C/A/T	< 0.001	P1070H	1000Genomes~ExAC~TOPMed	Missense Variant~Splice Region Variant	34	ENST00000274364.11
IQGAP2	rs200940839	5:76673589	C/A/T	< 0.001	P1070L	1000Genomes~ExAC~TOPMed	Missense Variant~Splice Region Variant	34	ENST00000274364.11
IQGAP2	rs140724393	5:76575751	A/G	< 0.001	Y97C	1000Genomes~ExAC~TOPMed~gnomAD	Missense Variant	31	ENST00000379730.7
IQGAP2	rs34968964	5:76665143	G/C	0.001	E833Q	1000Genomes~ESP~Phenotype_or_Disease~ExAC~TOPMed~gnomAD	Missense Variant	31	ENST00000379730.7
IQGAP2	rs34950321	5:76668682	C/T	0.005	T844I	1000Genomes~ESP~Phenotype_or_Disease~ExAC~TOPMed~gnomAD	Missense Variant~Splice Region Variant	32	ENST00000379730.7
IQGAP2	rs200940839	5:76673589	C/A/T	< 0.001	P1020H	1000Genomes~ExAC~TOPMed	Missense Variant~Splice Region Variant	34	ENST00000379730.7
IQGAP2	rs200940839	5:76673589	C/A/T	< 0.001	P1020L	1000Genomes~ExAC~TOPMed	Missense Variant~Splice Region Variant	34	ENST00000379730.7
IQGAP2	rs34968964	5:76665143	G/C	0.001	E379Q	1000Genomes~ESP~Phenotype_or_Disease~ExAC~TOPMed~gnomAD	Missense Variant	31	ENST00000396234.7
IQGAP2	rs34950321	5:76668682	C/T	0.005	T390I	1000Genomes~ESP~Phenotype_or_Disease~ExAC~TOPMed~gnomAD	Missense Variant~Splice Region Variant	32	ENST00000396234.7
IQGAP2	rs200940839	5:76673589	C/A/T	< 0.001	P566H	1000Genomes~ExAC~TOPMed	Missense Variant~Splice Region Variant	34	ENST00000396234.7
IQGAP2	rs200940839	5:76673589	C/A/T	< 0.001	P566L	1000Genomes~ExAC~TOPMed	Missense Variant~Splice Region Variant	34	ENST00000396234.7
IQGAP2	rs34968964	5:76665143	G/C	0.001	E379Q	1000Genomes~ESP~Phenotype_or_Disease~ExAC~TOPMed~gnomAD	Missense Variant	31	ENST00000502745.5
IQGAP2	rs34950321	5:76668682	C/T	0.005	T390I	1000Genomes~ESP~Phenotype_or_Disease~ExAC~TOPMed~gnomAD	Missense Variant~Splice Region Variant	32	ENST00000502745.5
IQGAP2	rs200940839	5:76673589	C/A/T	< 0.001	P566H	1000Genomes~ExAC~TOPMed	Missense Variant~Splice Region Variant	34	ENST00000502745.5
IQGAP2	rs200940839	5:76673589	C/A/T	< 0.001	P566L	1000Genomes~ExAC~TOPMed	Missense Variant~Splice Region Variant	34	ENST00000502745.5
IQGAP2	rs140724393	5:76575751	A/G	< 0.001	Y97C	1000Genomes~ExAC~TOPMed~gnomAD	Missense Variant	31	ENST00000505766.5
IQGAP2	rs34968964	5:76665143	G/C	0.001	E833Q	1000Genomes~ESP~Phenotype_or_Disease~ExAC~TOPMed~gnomAD	Missense Variant	31	ENST00000505766.5
IQGAP2	rs34950321	5:76668682	C/T	0.005	T844I	1000Genomes~ESP~Phenotype_or_Disease~ExAC~TOPMed~gnomAD	Missense Variant~Splice Region Variant	32	ENST00000505766.5
IQGAP2	rs200940839	5:76673589	C/A/T	< 0.001	P1020H	1000Genomes~ExAC~TOPMed	Missense Variant~Splice Region Variant	34	ENST00000505766.5
IQGAP2	rs200940839	5:76673589	C/A/T	< 0.001	P1020L	1000Genomes~ExAC~TOPMed	Missense Variant~Splice Region Variant	34	ENST00000505766.5
IQGAP2	rs34968964	5:76665143	G/C	0.001	E436Q	1000Genomes~ESP~Phenotype_or_Disease~ExAC~TOPMed~gnomAD	Missense Variant	31	ENST00000514001.5
IQGAP2	rs34950321	5:76668682	C/T	0.005	T447I	1000Genomes~ESP~Phenotype_or_Disease~ExAC~TOPMed~gnomAD	Missense Variant~Splice Region Variant	32	ENST00000514001.5
IQGAP2	rs140724393	5:76575751	A/G	< 0.001	Y120C	1000Genomes~ExAC~TOPMed~gnomAD	Missense Variant	31	ENST00000514350.5
SLIT2	rs547207452	4:20598286	G/A	< 0.001	G1208R	1000Genomes~ExAC~gnomAD	Missense Variant	32	ENST00000273739.9
SLIT2	rs547207452	4:20598286	G/A	< 0.001	G1187R	1000Genomes~ExAC~gnomAD	Missense Variant	32	ENST00000503823.5
SLIT2	rs547207452	4:20598286	G/A	< 0.001	G1191R	1000Genomes~ExAC~gnomAD	Missense Variant	32	ENST00000503837.5
SLIT2	rs547207452	4:20598286	G/A	< 0.001	G1195R	1000Genomes~ExAC~gnomAD	Missense Variant	32	ENST00000504154.6
SLIT2	rs547207452	4:20598286	G/A	< 0.001	G1105R	1000Genomes~ExAC~gnomAD	Missense Variant	32	ENST00000622093.4

*Indicates SIFT = deleterious; PolyPhen2 = probably damaging; CADD Class = likely deleterious; MetaLR Class = damaging; MutationAssesor Class = high

REVEL Class = likely disease causing

### *In-silico* transcript level functional impacts analysis of rare nsSNPs variants

We retrieved 36 transcript-level high-risk PTB-related nsSNPs in four genes, followed by further validation with six different algorithms to evaluate the functional impacts (Table 3). Missense variant rs532147352 of CNN1 found in 4 different transcripts have same amino acid variation (R>H) at four residue position according to their sequence length. PANTHER predicted all four transcript-level variations as ‘probably damaging’ with higher PhD-SNP probabilities. The disease associated PROVEAN, SNPs&GO, SNAP2, PMut and MutPred2 scores further classified these mutations as high-risk nsSNPs. In COL24A1, a single identified rs184448337, G>R had found associated with possibly damaging effects by PANTHER, with higher disease probabilities computed by PhD-SNP, PROVEAN, SNPs&GO, SNAP2, PMut and MutPred2 algorithms. In IQGAP2, rs140724393, Y>C with three different residue positions in 4 different transcripts according to the variability in sequence length were found associated with higher probabilities of pregnancy risks. Another SNP, i.e., rs200940839 have 5 different transcripts with four different residue-based variations (P>L/H) is regarded as higher disease-risk association probabilities. Third SNP rs34950321 exhibited in 6 different transcripts of the same substitution (T>I) of with 4 residue-level numbering had also found disease association. The last filtered variant of IQGAP2 was rs34968964 with six different transcripts. PATNHER predicted as probably damaging, while the rest of others were predicted as ‘neutral’ mutation, with least functional impact on protein (Table 3). For SLIT2, the rs547207452 occurred in 5 different transcripts with five residue positions of G>R substitution computed as probably damaging and pathogenic in functional impacts except SNP&GO probabilities (Table 3).

**Table 3:** Transcript sequence-based functional impact analyses of rare nsSNPs of PTB-related genes.

Gene	rsID	chr:Location	Amino Acid Change (Transcript Number)	PANTHER-PSEP Preservation Time	PhD-SNP	PROVEAN	SNPs&GO	SNAP2	PMut	MutPred2
CNN1	rs532147352	19:11549378	R186H (Tr1)	1237	0.793	-4.423	0.702	64	0.76	0.84
			R136H (Tr2)		0.769	-4.423	0.681	48	0.67	0.806
			R42H (Tr3)		0.67	-4.732	0.611	49	0.83	0.9
			R136H (Tr4)		0.769	-4.284	0.681	48	0.67	0.806
COL24A1	rs184448337	1:85842393	G1155R (Tr5)	362	0.934	-6.497	0.804	87	0.83	0.922
IQGAP2	rs140724393	5:76575751	Y147C (Tr6)	1500	0.827	-7.627	0.752	46	0.82	0.872
			Y97C (Tr7)		0.836	-7.627	0.76	16	0.56	0.842
			Y97C (Tr10)		0.835	-7.627	0.76	46	0.75	0.852
			Y120C (Tr12)		0.79	-8.107	0.724	49	0.79	0.83
	rs200940839	5:76673589	P1070H (Tr6)	1628	0.888	-7.695	0.843	71	0.82	0.903
			P1070L (Tr6)		0.925	-8.582	0.88	73	0.82	0.924
			P1020H (Tr7)		0.886	-7.695	0.842	48	0.8	0.89
			P1020L (Tr7)		0.924	-8.582	0.88	58	0.84	0.932
			P566H (Tr8)		0.88	-8	0.839	80	0.91	0.889
			P566L (Tr8)		0.92	-8.921	0.878	88	0.91	0.926
			P566H (Tr9)		0.88	-8	0.839	57	0.91	0.889
			P566L (Tr9)		0.92	-8.921	0.878	53	0.91	0.926
			P1020H (Tr10)		0.885	-7.695	0.841	30	0.9	0.896
			P1020L (Tr10)		0.924	-8.582	0.879	55	0.91	0.929
	rs34950321	5:76668682	T894I (Tr6)	1500	0.773	-5.295	0.537	60	0.73	0.57
			T844I (Tr7)		0.769	-5.295	0.533	31	0.75	0.45
			T390I (Tr8)		0.756	-5.299	0.521	16	0.77	0.483
			T390I (Tr9)		0.756	-5.299	0.521	59	0.89	0.472
			T844I (Tr10)		0.772	-5.295	0.535	46	0.79	0.483
			T447I (Tr11)		0.796	-5.172	0.542	16	0.79	0.459
	rs34968964	5:76665143	E883Q (Tr6)	1237	0.351	-2.796	0.313	33	0.45	0.538
			E833Q (Tr7)		0.341	-2.796	0.305	46	0.25	0.287
			E379Q (Tr8)		0.325	-2.664	0.294	44	0.37	0.297
			E379Q (Tr9)		0.325	-2.664	0.294	10	0.4	0.291
			E833Q (Tr10)		0.338	-2.796	0.303	52	0.44	0.306
			E436Q (Tr11)		0.623	-2.759	0.5	-3	0.68	0.274
SLIT2	rs547207452	4:20598286	G1208R (Tr13)	910	0.783	-7.461	0.494	68	0.51	0.847
			G1187R (Tr14)		0.781	-7.461	0.491	62	0.33	0.846
			G1191R (Tr15)		0.781	-7.461	0.492	64	0.74	0.831
			G1195R (Tr16)		0.776	-7.461	0.485	69	0.69	0.928
			G1105R (Tr17)		0.782	-7.461	0.495	66	-	0.865

### nsSNPs impact on protein stability, evolutionary conservation, and variation associated with structural and thermal properties of PTB-related proteins

**Fig 5:**
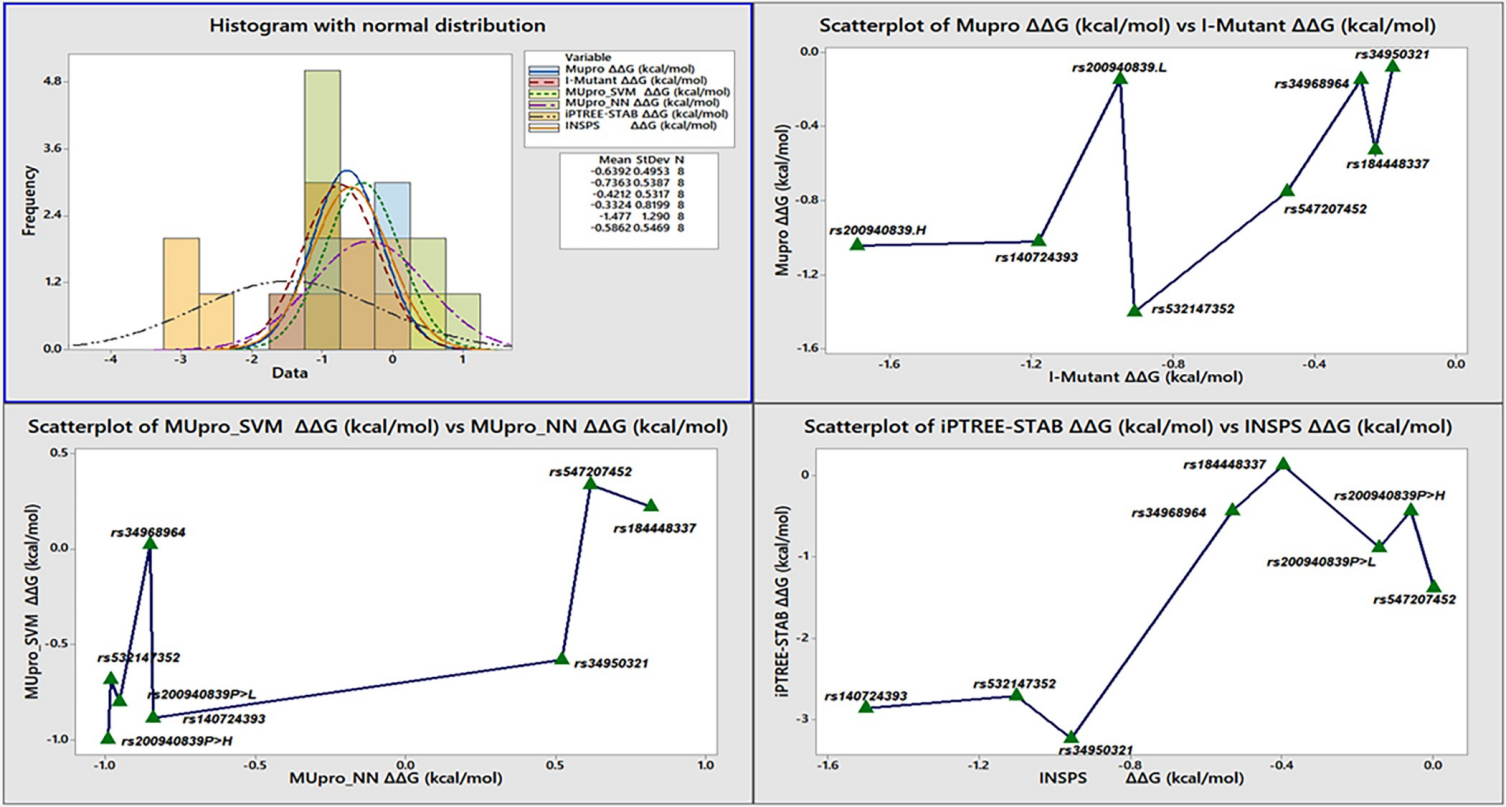
Histogram and scatterplot of rare nsSNPs of PTB-related gene mutations in comparison with functional impact analyses. The top-left section describes the mean and standard deviation values of various *in-silico* algorithms used to calculate the impact of mutations on protein stability using a normal distribution pattern. The remaining sections compared scatterplots between *in-silico* algorithms to determine how pathogenic nsSNPs in PTB-related genes affect protein stability.

nsSNP rs532147352 of CNN1 with all four transcripts showed the similar output about the structural and functional changes of a protein. This SNP result in the large decrease in the stability of protein computed by MUpro, I-Mutant, iPTREE and INSPS based on integrated predictor for protein stability change upon single mutation (R>H) (Fig 5 and Table 4). The ConSurf analysis predicted that the nsSNP of CNN1 gene (in all four transcripts) was highly conserved and exposed with highest score ranged between 8–9. The SOPMA secondary structural prediction characterized the residues position in random coil region of protein. The all four transcripts have highest proportion of coil (69–79%), with 43–64% of residues with few beta-strands and were exposed to surface (Table 5). This mutation causes the change of charge from positive to neutral with increase hydrophobicity of protein. The HOPE prediction showed that the mutant residue was smaller than the wild-type residue; this might lead to loss of interactions. Mutant residue was found near a highly conserved position, the mutation was located within a stretch of residues that was repeated in the protein as “Calponin-like 1”. This mutation might disturb this repeat and consequently any function other this repeat performed.

**Table 4:** Transcript-based computation of changes in free energy of amino acids of rare nsSNPs of PTB-related genes.

Gene	rsID	Amino Acid Change (Transcript Number)	MUpro ΔΔG (kcal/mol)	MUpro_SVM^a^ ΔΔG (kcal/mol)	MUpro_NN^b^ ΔΔG (kcal/mol)	I-Mutant ΔΔG (kcal/mol)	RI (SVM2/ SVM3)^c^	iPTREE-STAB ΔΔG (kcal/mol)	INPS ΔΔG (kcal/mol)
CNN1	rs532147352	R186H (Tr1)	-1.402	-0.6833	-0.979	-0.91	7 (D/LDS)	-2.7133	-1.10118
		R136H (Tr2)							-1.10118
		R42H (Tr3)							-1.09851
		R136H (Tr4)							-1.10118
COL24A1	rs184448337	G1155R (Tr5)	-0.524	0.2222	0.8167	-0.23	2 (D/DS)	0.1313	-0.397338
IQGAP2	rs140724393	Y147C (Tr6)	-1.023	-0.888	-0.8405	-1.18	3 (D/LDS)	-2.865	-1.49983
		Y97C (Tr7)							-1.49983
		Y97C (Tr10)							-1.51899
		Y120C (Tr12)							-1.55085
	rs200940839	P1070H (Tr6)	-1.043	-1	-0.9899	-1.69	9 (D/LDS)	-0.4333	-0.0594089
		P1070L (Tr6)	-0.1473	-0.8	-0.9517	-0.95	7 (D/LDS)	-0.89	-0.143668
		P1020H (Tr7)	-1.043	-1	-0.9898	-1.69	9 (D/LDS)	-0.433	-0.388285
		P1020L (Tr7)	-0.1473	-0.8	-0.9517	-0.95	7 (D/LDS)	-0.89	-0.436181
		P566H (Tr8)	-1.043	-1	-0.9899	-1.69	9 (D/LDS)	-0.433	-0.388285
		P566L (Tr8)	-0.1473	-0.8	-0.951	-0.95	7 (D/LDS)	-0.89	-0.492652
		P566H (Tr9)	-1.043	-0.8847	-0.9068	-1.69	9 (D/LDS)	0.7467	-0.388285
		P566L (Tr9)	-0.1473	-0.8	-0.9517	-0.95	7 (D/LDS)	-0.89	-0.492652
		P1020H (Tr10)	-1.043	-1	-0.9899	-1.69	9 (D/LDS)	-0.433	-0.388285
		P1020L (Tr10)	-0.1473	-0.8	-0.9517	-0.95	7 (D/LDS)	-0.89	-0.492652
	rs34950321	T894I (Tr6)	-0.079	-0.582	0.5196	-0.18	2 (D/DS)	-3.233	-0.957975
		T844I (Tr7)							-1.59598
		T390I (Tr8)							-0.957975
		T390I (Tr9)							-0.957975
		T844I (Tr10)							-0.957975
		T447I (Tr11)							-1.00685
	rs34968964	E883Q (Tr6)	-0.1455	0.0225	-0.8502	-0.27	0 (D/DS)	-0.434	-0.530299
		E833Q (Tr7)							-0.984343
		E379Q (Tr8)							-0.530299
		E379Q (Tr9)							-0.530299
		E833Q (Tr10)							-0.580128
		E436Q (Tr11)	-0.85026	0.0225	-0.8502	-0.27	0 (D/DS)	-0.434	-0.580128
SLIT2	rs547207452	G1208R (Tr13)	-0.74956	0.3386	0.616	-0.48	2 (D/DS)	-1.3775	-
		G1187R (Tr14)							
		G1191R (Tr15)							
		G1195R (Tr16)							
		G1105R (Tr17)							

^a^SVM = Support Vactor Machine

^b^NN = Neural Network

^c^SVM2 (Support vector machine2) = (D) Decrease = I-Mutant values in negative, SVM3 (Support vector machine3) = (LDS) Large Decrease in stability = I-Mutant values less than -0.5; DS (Decrease in stability) values >-0.5 = 0

**Table 5:** Evolutionary conservation analyses and protein structure prediction of rare nsSNPs of PTB-related genes.

Gene	Variant ID	Amino Acid Change (Transcript Number)	Consurf Score/ BNC score (CI)	Consurf Analysis	SOPMA Predicting Secondary Structure	Secondary Structural Information	Surface Accessibility	Change of Charge	Hydrophobicity
CNN1	rs532147352	R186H (Tr1)	9/-1.217 (-1.359, -1.151)	Highly Conserved and Exposed	Random Coil	Alpha-helix = 27.6%, Beta-strand = 3.4% and Coil = 69%	Burried = 35.7% and Exposed = 64.3%	Positive > Neutral	Increases
		R136H (Tr2)	8/-0.751 (-0.931,-0.654)			Alpha-helix = 21.5%, Beta-strand = 1.25% and Coil = 77.3%	Burried = 38.5% and Exposed = 61.5%		
		R42H (Tr3)	8/-0.673 (-0.839,-0.587)			Alpha-helix = 5.3%, Beta-strand = 15.8% and Coil = 78.9%	Burried = 56.6% and Exposed = 43.4%		
		R136H (Tr4)	8/-0.751 (-0.931,-0.654)			Alpha-helix = 21.5%, Beta-strand = 1.2% and Coil = 77.3%	Burried = 38.5% and Exposed = 61.5%		
COL24A1	rs184448337	G1155R (Tr5)	9/-1.087 (-1.165,-1.058)	Highly Conserved and Exposed	Random Coil	Alpha-helix = 1.3%, Beta-strand = 7.5% and Coil = 91.2%	Burried = 33.6% and Exposed = 66.4%	Neutral > Positive	Decreases
IQGAP2	rs140724393	Y147C (Tr6)	8/-0.806 (-1.068,-0.680)	Burried	Extended Strand	Alpha-helix = 68.9%, Beta-strand = 2.5% and Coil = 28.6	Burried = 40.5% and Exposed = 59.5%	-	Increases
		Y97C (Tr7)	8/-0.63 (-0.950,-0.476)			Alpha-helix = 69.9%, Beta-strand = 2.4% and Coil = 27.7%	Burried = 40.7% and Exposed = 59.3%		
		Y97C (Tr10)	7/-0.477 (-0.867,-0.267)			Alpha-helix = 71%, Beta-strand = 1% and Coil = 28%	Burried = 40.9% and Exposed = 59.1%		
		Y120C (Tr12)	8/-0.862 (-1.138,-0.713)			Alpha-helix = 76.1%, Beta-strand = 0.6% and Coil = 23.3%	Burried = 42.5% and Exposed = 57.5%		
	rs200940839	P1070H (Tr6)	7/-0.606 (-0.814,-0.465)	Exposed	Random Coil	Alpha-helix = 68.9%, Beta-strand = 2.5% and Coil = 28.6	Burried = 40.5% and Exposed = 59.5%	-	-
		P1070L (Tr6)	7/-0.606 (-0.814,-0.465)			Alpha-helix = 68.9%, Beta-strand = 2.5% and Coil = 28.6	Burried = 40.5% and Exposed = 59.5%		
		P1020H (Tr7)	9/-0.877 (-1.021,-0.787)	Highly Conserved and Burried		Alpha-helix = 69.9%, Beta-strand = 2.4% and Coil = 27.7%	Burried = 40.7% and Exposed = 59.3%		
		P1020L (Tr7)	9/-0.877 (-1.021,-0.787)			Alpha-helix = 69.9%, Beta-strand = 2.4% and Coil = 27.7%	Burried = 40.7% and Exposed = 59.3%		
		P566H (Tr8)	8/-0.746 (-0.947,-0.613)			Alpha-helix = 67.2%, Beta-strand = 3.7% and Coil = 29%	Burried = 41.4% and Exposed = 58.6%		
		P566L (Tr8)	8/-0.746 (-0.947,-0.613)			Alpha-helix = 67.2%, Beta-strand = 3.7% and Coil = 29%	Burried = 41.4% and Exposed = 58.6%		
		P566H (Tr9)	8/-0.795 (-0.958,-0.680)	Burried		Alpha-helix = 67.3%, Beta-strand = 4% and Coil = 28.7%	Burried = 41.3% and Exposed = 58.7%		
		P566L (Tr9)	8/-0.795 (-0.958,-0.680)			Alpha-helix = 67.3%, Beta-strand = 4% and Coil = 28.7%	Burried = 41.3% and Exposed = 58.7%		
		P1020H (Tr10)	8/-0.71 (-0.867,-0.587)			Alpha-helix = 71%, Beta-strand = 1% and Coil = 28%	Burried = 40.9% and Exposed = 59.1%		
		P1020L (Tr10)	8/-0.71 (-0.867,-0.587)			Alpha-helix = 71%, Beta-strand = 1% and Coil = 28%	Burried = 40.9% and Exposed = 59.1%		
	rs34950321	T894I (Tr6)	9/-1.062 (-1.130,-1.036)	Highly Conserved and Exposed	Random Coil	Alpha-helix = 68.9%, Beta-strand = 2.5% and Coil = 28.6%	Burried = 40.5% and Exposed = 59.5%	-	Increases
		T844I (Tr7)	9/-1.124 (-1.166,-1.120)			Alpha-helix = 69.9%, Beta-strand = 2.4% and Coil = 27.7%	Burried = 40.7% and Exposed = 59.3%		
		T390I (Tr8)	9/-1.097 (-1.174,-1.055)			Alpha-helix = 67.2%, Beta-strand = 3.7% and Coil = 29%	Burried = 41.4% and Exposed = 58.6%		
		T390I (Tr9)	9/-1.165 (-1.233,-1.128)			Alpha-helix = 67.3%, Beta-strand = 4% and Coil = 28.7%	Burried = 41.3% and Exposed = 58.7%		
		T844I (Tr10)	9/-1.046 (-1.145,-1.016)			Alpha-helix = 71%, Beta-strand = 1% and Coil = 28%	Burried = 40.9% and Exposed = 59.1%		
		T447I (Tr11)	9/-1.15 (-1.188,-1.144)			Alpha-helix = 88.4% and Coil = 11.6%	Burried = 43.2% and Exposed = 56.8%		
	rs34968964	E883Q (Tr6)	9/-1.029 (-1.130,-1.002)	Highly Conserved and Exposed	Alpha Helix	Alpha-helix = 68.9%, Beta-strand = 2.5% and Coil = 28.6%	Burried = 40.5% and Exposed = 59.5%	Negative > Neutral	Increases
		E833Q (Tr7)	9/-0.922 (-1.088,-0.950)			Alpha-helix = 69.9%, Beta-strand = 2.4% and Coil = 27.7%	Burried = 40.7% and Exposed = 59.3%		
		E379Q (Tr8)	9/-0.976 (-1.087,-0.907)			Alpha-helix = 67.2%, Beta-strand = 3.7% and Coil = 29%	Burried = 41.4% and Exposed = 58.6%		
		E379Q (Tr9)	9/-0.996 (-1.128,-0.917)			Alpha-helix = 67.3%, Beta-strand = 4% and Coil = 28.7%	Burried = 41.3% and Exposed = 58.7%		
		E833Q (Tr10)	9/-1.045 (-1.145,-1.016)			Alpha-helix = 71%, Beta-strand = 1% and Coil = 28%	Burried = 40.9% and Exposed = 59.1%		
		E436Q (Tr11)	9/-1.136 (-1.188,-1.114)			Alpha-helix = 88.4% and Coil = 11.6%	Burried = 43.2% and Exposed = 56.8%		
SLIT2	rs547207452	G1208R (Tr13)	9/-1.48 (-1.23, -1.122)	Highly Conserved and Burried	Beta Turn	Alpha-helix = 3.4%, Beta-strand = 12.8 and Coil = 83.7%	Burried = 36.4% and Exposed = 63.6%	Neutral > Positive	Decreases
		G1187R (Tr14)	9/-1.161 (-1.238, -1.129)		Random Coil	Alpha-helix = 3.7%, Beta-strand = 13.8 and Coil = 82.5%	Burried = 36.6% and Exposed = 63.4%		
		G1191R (Tr15)	9/-1.058 (-1.183, -1.014)		Beta Turn	Alpha-helix = 3.5%, Beta-strand = 14 and Coil = 82.6%	Burried = 36.5% and Exposed = 63.5%		
		G1195R (Tr16)	9/-1.146 (-1.231, -1.122)		Random Coil	Alpha-helix = 4.4%, Beta-strand = 12.6 and Coil = 83%	Burried = 37.3% and Exposed = 62.7%		
		G1105R (Tr17)	9/-1.056 (-1.190, -0.977)		Beta Turn	Alpha-helix = 2.8%, Beta-strand = 13.6 and Coil = 83.7%	Burried = 36.1% and Exposed = 63.9%		

In COL24A1, the nsSNP rs184448337 (G>R) mutation also exhibited the decrease in stability of protein, as computed by the relevant software, which might affect the function and associated with disease occurrence (Fig 5 and Table 4). The SNP of COL24A1 possessed highest ConSurf score *(9*) located in random coil region of protein and were highly conserved and exposed (Table 5). The alpha-helix and beta-strands were least in proportion as compared to coil which was 91.2% and 66.4% of residues were exposed to surface (Table 5). The mutation results in the change of charge from neutral to positive which decrease the hydrophobicity. HOPE inference predicted that the mutant residue was bigger than the wild-type residue. The wild-type residue was more hydrophobic than the mutant residue. The mutation is found within a domain, annotated in UniProt as "Collagen-like 11". The mutation introduces an amino acid with different properties, which can disturb this domain and abolish its function. The wild-type residue was a G, the most flexible of all residues. This flexibility might be necessary for the protein’s function. Mutation of this G with R can obliterate this function.

In IQGAP2 gene, the nsSNP rs140724393 resulted in the large destabilization of protein structure due to Y>C mutation, which might lead to severe pathophysiological consequences (Fig 5 and Table 4). This mutation identified in all four transcripts was also found conserved, buried, and located in the extended strand of protein secondary structure. The higher secondary structural proportion was alpha-helix (68.9–76%) and around 59% of residues were exposed to surface (Table 5). HOPE inference predicted that the mutant residue is smaller than the wild-type residue. The mutant residue was more hydrophobic than the wild-type residue. This can result in loss of hydrogen bonds and/or disturb correct folding. The mutation is located within a domain annotated in UniProt as "Calponin-homology (CH)". The mutation introduces an amino acid with different properties which can disturb this domain and abolish its function. The mutated residue was in a domain that is important for binding of other molecules. Mutation of the residue might disturb this function.

rs200940839 through various analytical tools was predicted as higher protein structural changes which could resulted in the destabilization of protein function (Fig 5 and Table 4). The mutation rs200940839 (P>H, L) identified in all five transcripts were found conserved, buried, and located in random coil, except (P1070H/L) in transcript ENST00000274364.11, which found exposed and moderately conserved. The higher proportion of secondary structure was alpha-helix (~69%) and around 59% of residues were exposed to surface (Table 5). The mutant residue is bigger than the wild-type residue. The wild-type residue is more hydrophobic than the mutant residue which is proline. The mutation is located within a domain annotated in UniProt as "Ras-GAP". The mutation introduces amino acids with different properties which can disturb this domain and abolish its function. Prolines are known to be very rigid and therefore induce a special backbone conformation which might be required at this position. The mutation can disturb this special conformation. The mutated residue is located on the surface of a domain with unknown function. The mutation might cause loss of hydrophobic interactions with other molecules on the surface of protein.

The mutation rs34950321 (T>I) was predicted to cause higher protein structural changes which could resulted in the destabilization of protein function (Fig 5 and Table 4). This mutation was identified in six transcript that showed highly conserved, exposed area and present in the random coil region. The highest secondary structural proportion was alpha-helix (~68–88%) and ~58% of residues were exposed to the surface (Table 5). According to the HOPE inference, the mutant residue was bigger than the wild-type residue and was more hydrophobic than the wild-type residue. The residue is buried in the core of a domain. The differences between the wild-type and mutant residue might disturb the core structure of this domain. The hydrophobicity of the wild-type and mutant residue differs. The mutation will cause loss of hydrogen bonds in the core of the protein and as a result disturb correct folding.

The mutation rs34968964 (E>Q) in all six transcripts were found highly conserved, exposed, and located in alpha-helix region of protein (Fig 5 and Table 4). Around 68–88% residues were alpha-helix with ~59% of residues were exposed to surface (Table 5). The HOPE inferred that the mutation converts the wild-type residue in a residue that does not prefer α-helices as a secondary structure. The charge was changed from negative to neutral and the mutated residue was located on the surface of a domain with unknown function. The residue was not found to be in contact with other domains of which the function is known within the used structure. However, contact with other molecules or domains will still possible but might be affected by this mutation. The charge of the wild-type residue was lost by this mutation and hydrophobicity increases. This can cause loss of interactions with other molecules. The protein stability tools also predicted the decrease in the stability of structure and function, as indicated by various energetic-based *in silico* estimation.

In SLIT2 gene, the mutation rs547207452 (G>R) in all five transcripts was not computed by ConSurf software, however, transcript IDs ENST00000273739.9, ENST00000503837.5, ENST00000622093.4 were in beta-turns and the rest two were with random coil region as this protein has >82% coil in secondary structure distribution. The ~63% residues were exposed to surface, and mutation results in decreasing the hydrophobicity (Table 5). The HOPE inference predicted that the mutant residue was bigger than the wild-type residue. The mutation was located within a domain annotated in UniProt as "Laminin G-like". The mutation introduces an amino acid with different properties which can disturb this domain and abolish its function. The wild-type residue is a G, the most flexible of all residues. This flexibility might be necessary for the protein’s function. Mutation of this G can abolish this function which was also clear from the computation of various indicators to investigate the impact of mutation on protein function, leading towards disease progression.

### GTEx investigation of PTB gene/proteins in female reproductive tissues

GTEx database identified the CNN1 Isoform 2 as the highly expressed gene in female reproductive tissues when compared against the pattern of gene expression to the rest of all 4 genes (S1 Table). The GTEx analysis for CNN1 showed bladder (n = 7) median TPM of 706, cervix-ectocervix (n = 9) 302, cervix-endocervix (n = 10) 315.9, fallopian tubes (n = 9) 986.8, ovary (n = 180) 44.08, uterus (n = 142) 1385, vagina (n = 156) 191.8, respectively. The rest three (IQGAP2, COL24A1& SLIT2) genes showed the lowest comparative indices as compared with CNN1 (S2 Table).

### Protein structural coverage identified genes expressed in female reproductive tissues

The protein-wise structural coverage of selected proteins was extracted from the PDB. The highest protein structural coverage of CNN1 was 41% with amino acids length of 136 residues positioned at 20–142 through NMR method (Table 6). However, IQGAP2 and SLIT2 have least coverage, 6–24% and 7–12%, respectively with existing templates. COL24A1 has no structural coverage with PDB templates (Table 6).

**Table 6:** PDB structural coverage of identified PTB-related genes.

Gene Name	Protein Name	UniProt ID	PDB Structure	PDB Entry	Length (amino acids)	Coverage (%)	Position	Method	Resolution (Å)	Chain	Theoretical weight (KDa)
CNN1 (calponin 1)	calponin1 isoform 2	P51911	Partial Present	1WYP	136	41	20–142	NMR	Not mentioned	A	14.99
COL24A1 (collagen type XXIV alpha 1 chain)	collagen alpha—1(XXIV) chain isoform 2	Q17RW2	Not Present	N/A	N/A	N/A	N/A	N/A	N/A	N/A	N/A
IQGAP2 (IQ motif containing GTPase activating protein 2)	ras GTPase—activating—like protein IQGAP2 isoform 3, 2 and 4	Q13576	Present (partial and multiple)	3IEZ	114	6	1476–1571	X-ray	1.5	A/B	13.23
				4EZA	114	6	1476–1571	X-ray	1.5	A/B	13.23
				5CJP	387	24	875–1258	X-ray	2.6	E/F	44.06
SLIT2 (slit guidance ligand 2)	slit homolog 2 protein isoform 2 & 3 precursor	O94813	Present (partial and multiple)	2V70	220	14	504–714	X-ray	3.01	A/B/C/D	24.89
				2V9S	220	14	271–480	X-ray	2	A/B/C/D	24.71
				2V9T	117	7	271–479	X-ray	1.7	B	13.1
				2WFH	193	12	726–907	X-ray	1.8	A/B	21.46

### Homology modeling of CNN1 isoform 2

The secondary structure details of CNN1 isoform 2 showed that 29.60% (82 residues) formed alpha-helix, 14.80% (41 residues) were involved in beta-strand, 7.58% (21 residues) were involved in beta-turn and random coil were 48.01% (133 residues). CNN1 isoform 2 was found to be the highly expressed protein (in female reproductive tissues) with maximum structural coverage available on PDB database (Table 6). Therefore, the template selection for the homology modeling of CNN1 isoform 2 was carried by BLAST tool (Table 7). Top 5 alignment descriptions were used in which the highest similarity was seen with the solution structure of CH domain of human calponin 1 isoform 1, PDB ID: 1WYP (43% query coverage, E value 2.00×10^−86^, and 94% identity) (Table 8). Afterwards, homology modeling was carried out by PHYRE2 the 3^rd^ ranked structure was identical with the BLAST high similarity results of CNN1 isoform 2 (Table 8). The 3D stereochemical analysis and validation of 3D model of CNN1 was evaluated by the quality factor of ERRAT (73.68%) and 97 residues lied in the most favored region (S3 Table). Only 10 residues were in additional allowed region while no residue was found in the generously allowed or disallowed region. The Verify3D score was 83.74% which means that >80% residues have averaged 3D-1D score > = 0.2. Numbers of non-glycine and non-proline residues were 108 (glycine = 10 and proline = 2). The residual properties showed that maximum deviation observed was 2.6, the bond length/angle was 4.8, and the bond contact was 14. The G-factor analyses showed that dihedrals score was -0.01, covalent score was 0.69 with overall score of 0.26. Planar groups of 3D model showed that 100% were within the limit (S3 Table). The CNN1 isoform 2 exhibited a Calponin homology (CH) domain started at R10 and ended at L107. The low complexity region (LCR) of CNN1 was observed in two regions which are K114-K123 and A265-N275, respectively.

**Table 7:** Protein-BLAST alignment of CNN1 isoform 2 sequence.

S. No.	Alignment Description	Scientific Name	Maximum Score	Total Score	Query Coverage	E -value	Identity (%)	Coverage Length	PDB ID
**1**	Solution structure of the CH domain of Calponin 1	*Homo sapiens*	255	255	0.43	2.00×10^−86^	100	136	1WYP
**2**	NMR Structure of the CH Domain of Calponin	*Gallus gallus*	189	189	0.38	4.00×10^−61^	81.13	108	1H67
**3**	Solution structure of the CH domain of calponin-2	*Homo sapiens*	182	182	0.46	7.00×10^−58^	67.18	146	1WYN
**4**	Solution structure of the CH domain of transgelin-2	*Homo sapiens*	86.3	86.3	0.43	2.00×10^−20^	37.21	155	1WYM
**5**	Solution structure of the CH domain of Rho guanine nucleotide exchange factor 6	*Homo sapiens*	57	57	0.26	6.00×10^−10^	40	121	1WYR

**Table 8:** Top 5 templates of CNN1 isoform 2 by PHYRE2 tool.

Rank	Template	PDB Title	PDB Header	Chain	Molecule	Number of Aligned Residues	Identity (%)	Confidence (%)
**1**	1WYN	Solution structure of the ch domain of human Calponin-2	Structural Protein	A	Calponin-2	133	66	100
**2**	1WYM	Solution structure of the ch domain of human Transgelin-2	Structural Protein	A	Transgelin-2	130	38	100
**3**	1WYP	Solution structure of the ch domain of human Calponin 1	Structural Protein	A	Calponin 1	123	94	100
**4**	2RR8	Solution structure of calponin homology domain of IQGAP1	Protein Binding	A	IQGAP1 protein	147	20	100
**5**	3I6X	Crystal structure of the calponin homology domain of IQGAP1	Calmodulin-binding, Membrane Protein	C	ras gtpase-activating-like protein IQGAP1	129	16	1

Finally, we selected the highest percent similarity (94%) model of 123 aligned residues as our target protein for further investigation of this work (Table 8). For subsequent comparison with generated homology model through PHYRE2 tool, we downloaded the crystal structure of 1WYP from PDB database as template. The overall RMSD (root mean square deviation) value of homology model was 2.21 obtained through its superimposition with PDB 3D-template *i*.*e*., 1WYP. However, RMSD value for α-carbons was 2.27, back-bone atoms were 2.22 and heavy atoms were 2.35.

### Prediction and validation of binding cavities by consensus mode

The binding cavities were validated CB-dock approach. The highest binding affinity (docking score) was examined through the *in-silico* interaction of homology model (CNN1 Isoform 2) with progesterone in cavity-graded pose rank 1 (-6.5 kcal/mole) (S4 Table). The detailed analysis of amino acids interaction was explored through 3D and 2D visualization of cavities interaction which reveals the interaction of R10, D84, S102, L105, K123 and Y124 (S4 Table).

### Selection and screening of drug molecules involved in PTB management

From DrugBank database, initially 17 drugs were extracted as small drug molecules (S5 Table). All 17 drugs were screened in relevance with the drug targets information and their mode of action, ultimately reduced to 7 PTB-related drugs. The Lipinski’s rule of five was then applied on all 7 screened drug molecules, 5 drugs fulfilled the completely criteria (S6 Table).

### *In-Silico* molecular mechanics and energetic estimations of filtered PTB-related drugs interacted with CNN1 isoform 2

**Fig 6:**
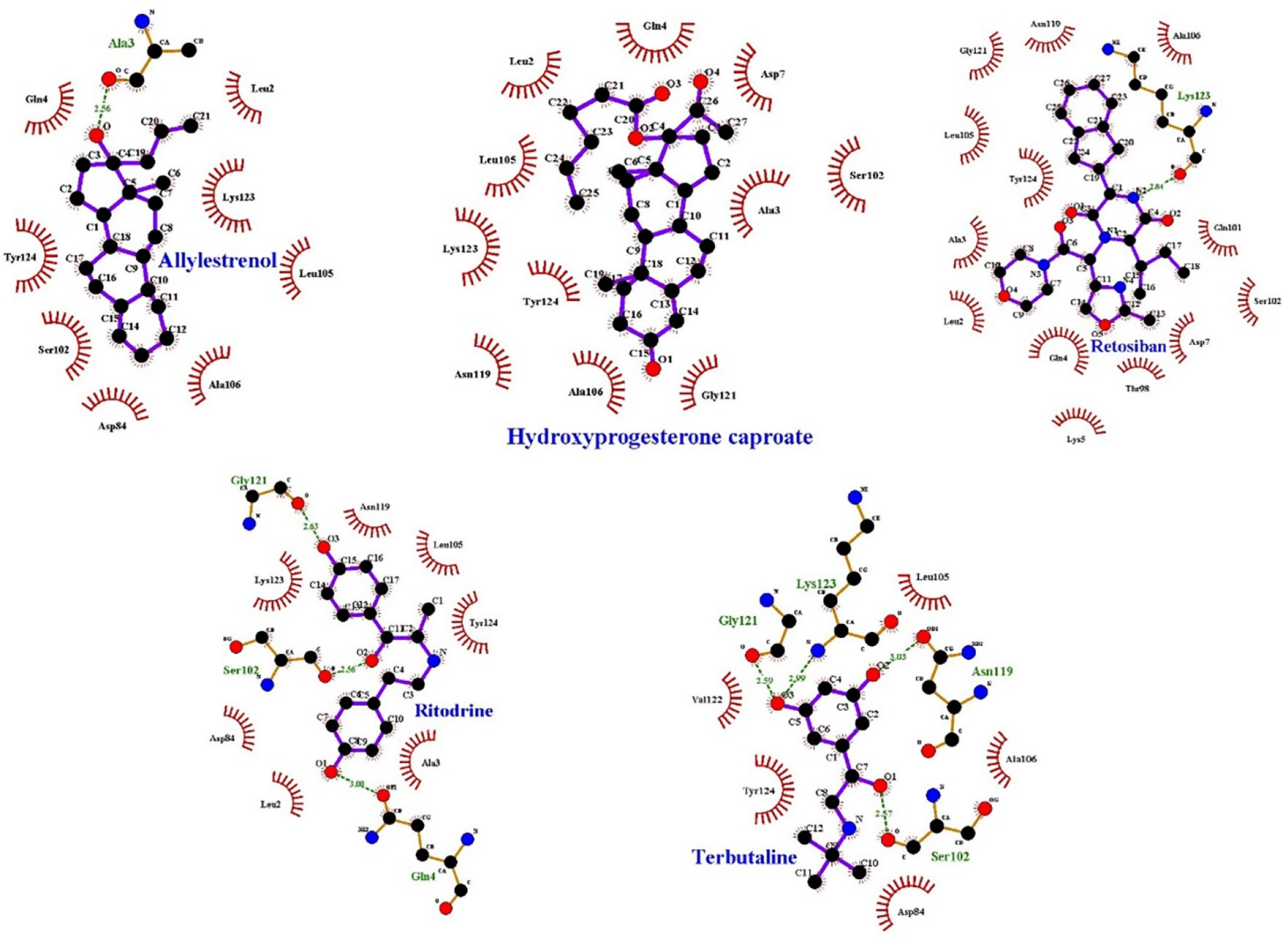
2D Ligplot showing molecular docking interactions of CNN1 isoform 2 with PTB-drugs. Protein-ligand based graphical depiction showing the potential interacting residues of CNN1 protein with Allylestrenol, Hydroxyprogesterone caproate, Retosiban, Ritodrine and Terbutaline.

The identified residues through CB dock approach were selected as key interacting residues and further used for *in-silico* analyses of all five drugs interactions with homology model (CNN1). The estimated energies and molecular mechanics of all five drugs showed a significant binding interaction with homology model (Table 9 and Fig 6). The highest binding energy was estimated with Retosiban (-9.43 kcal/mol), followed by Hydroxyprogesterone caproate (-8.19 kcal/mol). Allylestrenol and Ritodrine were also found with high binding energy, -7.56 and -7.39 kcal/mol, respectively.

**Table 9:** Docking-guided molecular mechanics and estimation of ligand binding energies of PTB-related drugs with CNN1 isoform 2.

S. No.	Drugs	ΔE_vdw_	ΔE_ele_	ΔE_MM_	ΔE_(unbound)_	ΔE_(torsional)_	ΔE_(Total Internal)_	ΔE_(intermolecular)_	ΔG_bind_ (kcal/mol)	Inhibition Constant (*K*_*i*_)
**1**	Allylestrenol	-8.33	-0.05	-8.38	-0.2	0.82	-0.2	-8.39	-7.56	2.89 uM
**2**	Hydroxyprogesterone caproate	-10.29	0.18	-10.11	-0.65	1.92	-0.65	-10.11	-8.19	991.82 nM
**3**	Retosiban	-11.07	-0.01	-11.08	-2.05	1.65	-2.05	-11.07	-9.43	123.25 nM
**4**	Ritodrine	-9.61	-0.26	-9.87	-0.68	2.47	-0.68	-9.86	-7.39	3.81 uM
**5**	Terbutaline	-7.44	-1.35	-8.79	-0.33	1.92	-0.33	-8.8	-6.87	9.14 uM

The overall *in-silico* pharmacokinetics predictions of 5 screened PTB-related drugs showed safest therapeutic properties with regards to the basic physicochemical characteristics of absorption, distribution, metabolism, excretion and toxicity indices (S7 Table).

## Discussion

In this study we investigated the functional impact of 36 rare coding variants/rare-high risk nsSNPs through *in-silico* machine learning algorithms including Panther-PSEP, PhD-SNP, PROVEAN, SNP&GO, SNAP2, PMut and MutPred2 tools. Present findings through Panther-PSEP showed all 6 nsSNPs from CNN1, IQGAP2, and SLIT2 with the highest probability of deleteriousness, which may be related, as the longer a position has been preserved, the more likely that it will have a deleterious effect [57]. Interestingly, our results from PhD-SNP, SNPs&GO, PROVEAN, SNAP2, PMut, and MutPred2 showed all transcripts nsSNPs as pathogenic variants, which shows that these residue-based substitutions are associated with damaging effects on the biological function of PTB protein. However, there was some variation in a few transcript scores denoting lesser pathogenicity of mutations.

A genome-wide association study (GWAS) on Finnish population reported the occurrence of fetal SLIT2 genomic variation [58]. SLIT2 and ROBO1 gene expression in the cells of placenta and trophoblast may be linked with spontaneous PTB [58]. In this manner, variant annotations with elaborative approach regarding their role in PTB are needed to interpret the impact of point mutations which can lead to protein structural and functional changes, and may set the basis for the occurrence of APOs [59]. Our findings about point mutations that may influence protein stability can be useful in drug designing and prevention of PTB.

Predicting the effect of genomic variations on protein stability such as amino acid substitution is useful for screening of disease-causing SNPs [36] in proteins. Our findings reported the transcript-based computation of free energy changes of all seven rare nsSNPs of PTB-related genes having a decrement in protein stability, except the computed score of iPTREE-STAB of COL24A1 gene (rs184448337). This was further validated through ConSurf, SOPMA and HOPE tools which investigated these mutations, as they were highly conserved from the point of evolution.

CNN1, thin filament-associated protein known to regulate smooth muscle contraction, includes regulation, modulation of contraction processes, and activation of downstream pathways. It is reported to bind actin, calmodulin and tropomyosin [60, 61]. The regulatory roles in both cytoskeletal dynamics and signaling of CNN1 are due to its CH (calponin homology) domain [61]. The role of CH domain in the onset of some diseases like cancers and asthma were reported [61]. The 3D protein model that we generated in this work, namely CNN1, has been reported as a biomarker for the prediction of preterm birth among pregnancies complicated by threatened preterm labor, in addition to cervical length measurement [62, 63]. However, its protein functions and corresponding roles in diseases still needs to be explored, and this lack of literature will potentially link its contributing pathophysiological role in the onset of PTB. The identification of rare exonic variants as genetic markers for any disease with high relevance is a significant contributor for reporting the genetically associated risk of diseases [21]. All the four identified rare pathogenic variants of the CNN1 gene have pertinent amino acid substitution/variation observed beyond the reported structural coverage. This has been considered as an obstructing limitation of this work, which narrows the generalizability of the impact of the present results.

The current pharmacological basis for managing PTB with progesterone is well reported [64]. Unfortunately, the availability of information regarding the molecular or genomic basis of drug interactions to manage PTB (associated with multi-etiological factors) is not well documented and sets the stage for this research. Investigation of key active sites or interacting amino acids as potential targets (corresponding protein cavities) and their binding interactions with intervening bioactive compounds helps to explore PTB management challenges.

Drug target identification describes the process of identifying the direct molecular target available as protein or on nucleic acid, prominent for continuing interaction with usually small drug molecule [65]. An effective drug target needs to be therapeutically relevant to the disease phenotype and will not cause side effects by disrupting the homeostasis (physiological) mechanism/function of the target protein [65]. Hence, in this study the identification of potential druggable protein binding cavities showed the amino acids interaction (R10, D84, Q99, S102, L105, K123, Y124) with highest docking affinity, identified, confirmed through CB-dock [52] and COACH meta-server [53]. This strategy helps in the identification of PTB biomarker-related drugs interactions site present on our candidate gene target [65].

We selected five main drugs, including Hydroxyprogesterone caproate, Allylestrenol, Terbutaline, Ritodrine, and Retosiban with potential drug-likeness attributes, and performed residue specific molecular docking with our PTB target protein (CNN1 isoform 2). The obtained energetic estimate plays decisive role regarding the drug designing approach based on the binding interactions of PTB protein with therapeutic compound to identify protein targets through structural conformation held with non-covalent interactions [66]. The association of selected drugs used to manage PTB with the CNN1 isform2 protein 3D model showed an inhibitory impact on this target gene involved in PTB. The highest binding energetic estimation was observed in Hydroxyprogesterone caproate and Allylestrenol with increased attractive charge interaction at the D7 and D84 positions of Hydroxyprogesterone caproate. The commonest residues of CNN1 isoform 2 observed in the different kinds of chemical interactions of all five drugs were S102, L105, A106, K123, and Y124. The observed Pi-sigma interactions (Pi-alkyl and alkyl) may be due to large charge transfer involvement which helps in the intercalation of the binding of drugs with prominent amino acids of CNN1 isoform 2 [67]. The highest numbers of hydrogen bonds were formed in Ritodrine (Q4, S102, G121, K123) and Terbutaline (D84, S102, N119, G121, K123), respectively. However, Retosiban (K123) and Allylestrenol (A3) showed a single hydrogen bond interaction. This exhibits the strength of PTB protein-drug binding complex and its interaction with the possible therapeutic inhibitory action as it represents the non-covalent force of interaction (formed between various positively charged hydrogen atoms and the existing electron negative atom; nitrogen or oxygen-lone pairs of electrons) of the drug entity [68].

Drug discovery and drug development processes are preliminary linked with an *in-silico* investigation approach in assessing the pharmacokinetics (ADMET; absorption, distribution, metabolism, excretion, and toxicology) properties of the drug of interest. The *in-silico* screening markedly decreased the rate of attrition before subjecting the drug to clinical trials [56, 69].

The investigated physicochemical properties of Terbutaline, Ritodrine, and Retosiban showed high drug solubility properties, while Allylestrenol and Hydroxyprogesterone caproate were less soluble with elevated distribution coefficient values. The consideration of drug solubility plays a significant role in the selection of drug molecule, and its evaluation as best possible usage [56, 69]. Besides the molecular weight, the concept of hydrophobicity may affect the degree or extent of solubilization and drug interactions with compounds like bile acids, which are facial amphipathic as they contain both lipid soluble (hydrophobic) and hydrophilic (polar) faces, and are also critical for transport and absorption of fat-soluble compounds [69]. Similarly, high hydrophobicity was assessed in Hydroxyprogesterone caproate and Allylestrenol as the role of molecular hydrophobic interactions (the tendency of interaction of non-polar molecules in body fluids) cannot be ignored. The hydrophobic interaction is one of the major interacting molecular forces resulting from the diffusion of drug molecules through the cell (plasma) membrane and binds to the internally localized receptor for gene expression or target action [56, 69, 70]. This might serve as the basis to understand the binding of Hydroxyprogesterone caproate and Allylestrenol (both showed high docking/binding scores) with our CNN1 isoform 2.

After administration of the drug, the main focus of interest in drug discovery is the availability strength of drug into the bloodstream and then how fast the required magnitude of drug reaches its specific target site (receptor) for the purpose of therapeutic action [56, 69]. The Caco-2 (the human colon epithelial cancer cell line) permeability assay can be used to predict *in vivo* absorption of drugs, as it measures the quantification rate of a drug across polarized Caco-2 mono-layers [56, 69]. The *in-silico* outcome for Caco-2 permeability regarding human intestinal absorption of selected PTB-drugs showed the optimal range, further validated through HIA (human intestinal absorption) pharmacokinetic percentage scores [56, 69].

Bioavailability represents the administered drug fraction which reaches the systematic (bloodstream) circulation as a sub-category of drug absorption variable/factor [69]. Hydrophobic drugs are considered as the most favourable class of drug choice as they alter the plasma membrane fluidity to increase the passive trans-cellular drug permeation [70, 71]. This tendency also modulates the tight junction across the cell membrane to allow increased para-cellular diffusion of drug molecules [56, 69]. The *in-silico* pharmacokinetics for the bioavailability (F 30%) of five PTB drugs showed the highest values for Ritodrine (64.30%), Allylestrenol (48.20%), and Retosiban (44.50%). Terbutaline (26.20%) and hydroxyprogesterone caproate (19.30%) had lower range values. This generally strengthens our point of selection of small molecules and should be replicated in future novel drug compounds for finding best therapeutic role in relation for the management of PTB.

The investigational outcome regarding volume distribution of all five screened PTB-related drugs showed an evenly distributed pattern, and the drug Ritrodine is highly lipophilic in nature, among the rest. Except for Terbutaline, the plasma protein binding interaction of all four other drugs was satisfactory as it indicates the high binding affinity with CNN1 isoform 2, also cleared from the *in-silico* drug binding energetic estimates. When a drug enters the systematic (bloodstream) circulation, it is distributed to the different tissues of the body. The rate of drug entry into the tissues mainly depends on the rate of blood flow to the respective tissues and the various partition characteristics between blood and tissue. Drugs reach the CNS (central nervous system) through brain capillaries and CSF (cerebrospinal fluid), but the restriction of drug penetration to this vicinity is mainly regulated by the brain’s permeability characteristics [56, 69]. Allylestrenol and Hydroxyprogesterone caproate showed the highest blood brain barrier (BBB+++) indications. This may be due to the rate of drug penetration into CSF and its further categorization by the extent of protein binding, degree of drug-ionization, and the lipid-water partition coefficient of the drug molecules [69]. The significance of the slow penetration rate of drugs in the brain is mainly associated with high protein binding and this is again validated in the case of allylestrenol and hydroxyprogesterone caproate.

When any drug or pharmacological compound is administered to the body by mouth, the first pass metabolism occurs in the liver tissues. This phenomenon typically occurs when a drug gets metabolized at a particular location in the body that ultimately results in the reduced active concentration of the drug upon reaching its target site of action or the systemic circulation. This pre-systemic metabolism may greatly reduce the bioavailability of the drug and serve as the basic justification for most of the drug’s withdrawal from the market, in addition to the reported drug-induced liver injury. Similarly, cytochrome P450 (CYP-450) serves as a super-family of proteins containing heme as a cofactor, which constitutes its significant role in metabolism and detoxification of xenobiotic [56, 69]. During the drug metabolism process, the quantity of drug required for effective concentration to exhibit therapeutic action should not be quickly metabolized by CYP-450. Hence, the CYP-450 enzyme is fundamentally used for understanding the metabolism of drugs through oxidizing substances by Fe (iron) and can metabolize a large variety of xenobiotic substances. CYP-450 can be inhibited or induced by the administration of certain drugs, which may lead to drug-drug interactions with unexpected adverse reactions or potential therapeutic failures [69]. Therefore, the substantial information about the drugs’ metabolism based on CYP-450 (the most potent inhibiting and inducing drugs) assessment can be beneficial in reducing the possible drug’s adverse reactions and interactions. Our present predicted pharmacokinetics findings related to selected PTB-drug metabolism showed the significant impact of all five therapeutic compounds.

The half-life time and clearance rate of any drug play a vital role in the drug development process as it involves the removal of drug/substance from the body either as bio-transformed metabolites or in unchanged form (intact drug form). This whole process is supported by a variety of factors which impact drug excretion, such as intrinsic drug properties (polarity, pH, and molecular mass) and may induce kidney tissue injuries [56, 69]. Moreover, LD50 is an important measure of drug toxicity; a drug with a small LD50 value is more toxic than a drug with a high LD50 value [56, 69]. The predicted pharmacokinetics results indicated the satisfactory value range for all five screened PTB-drugs.

In future, a follow up study may look for rationalization and optimization of PTB protein/biomarkers (obtained from the present effort) through *in-vitro* challenge by using variable levels of perturbing variants of PTB. Furthermore, this study will lay the groundwork for future research into the gene-associated quest to better understand the role of CNN1 protein, particularly its CH domain, specifically probing its relevance with PTB via functional genome-wide sequencing and detailed molecular analysis. Finally, the role of identified consensus interacting residues of this *in-silico* experimentation will be further tested with novel derivatives, which will pave the way for discovering new therapeutic compounds to manage the challenge of PTB.

## Conclusion

PTB is an important perinatal health issue and addressing this global health problem through *in-silico* genes-based strategies not only developed a better understanding to improve access to cost effective obstetric and neonatal care but also reduce the neonatal and childhood mortality. This research work comprising the *in-silico* transcript-based analyses of nsSNPs of CNN1, COL24A1, IQGAP2 and SLIT2 genes highlighted that 7 nsSNPs with 36 different transcripts have shown the most pathogenic among all known and reported nsSNPs. These nsSNPs possess marked energetic differences in terms of their impact on the stability of protein. The structural docking-based experimentation of CNN1 gene and its molecular interaction (S102, L105, A106, K123, Y124) analysis could serve as an intervention target for the prevention of PTB. Therefore, on a country level, if more efforts are made in terms of exploring novel genetic variations to identify non-synonymous pathogenicity from the same set of genes with further development and incorporation of *in-silico* tools will pave the way for managing PTB.

## Supporting information

S1 Table ALFA frequencies of rare nsSNPs of PTB-related genes. (XLSX)

S2 Table Gene expression details of female reproductive tissues. (XLSX)

S3 Table 3D Homology model assessment and validation of CNN1 isoform 2. (XLSX)

S4 Table Cavity-based blind docking and amino acids interactions of CNN1 isoform 2 with progesterone. (XLSX)

S5 Table List of PTB-related drugs extracted from DrugBank database. (XLSX)

S6 Table Shortlisted PTB-related drugs after manual inspection and applying Lipinski’s rule of 5 screening. (XLSX)

S7 Table *In-silico* predicted pharmacokinetics profile of screened PTB-related drugs. (XLSX)
